# Combining Data Independent Acquisition With Spike-In SILAC (DIA-SiS) Improves Proteome Coverage and Quantification

**DOI:** 10.1016/j.mcpro.2024.100839

**Published:** 2024-09-11

**Authors:** Anna Sophie Welter, Maximilian Gerwien, Robert Kerridge, Keziban Merve Alp, Philipp Mertins, Matthias Selbach

**Affiliations:** 1 Division of Proteome Dynamics, Max Delbrück Center for Molecular Medicine, Berlin, Germany; 2 Faculty of Life Sciences, Humboldt-Universität zu Berlin, Berlin, Germany; 3 Division of Proteomics, Max Delbrück Center for Molecular Medicine, Berlin, Germany; 4 Berlin Institute of Health, Core Unit Proteomics, Berlin, Germany; 5 Charité, Universitätsmedizin Berlin, Berlin, Germany

**Keywords:** SILAC, DIA, data independent acquisition, multispecies benchmark, spike-in

## Abstract

Data-independent acquisition (DIA) is increasingly preferred over data-dependent acquisition due to its higher throughput and fewer missing values. Whereas data-dependent acquisition often uses stable isotope labeling to improve quantification, DIA mostly relies on label-free approaches. Efforts to integrate DIA with isotope labeling include chemical methods like mass differential tags for relative and absolute quantification and dimethyl labeling, which, while effective, complicate sample preparation. Stable isotope labeling by amino acids in cell culture (SILAC) achieves high labeling efficiency through the metabolic incorporation of heavy labels into proteins *in vivo*. However, the need for metabolic incorporation limits the direct use in clinical scenarios and certain high-throughput experiments. Spike-in SILAC (SiS) methods use an externally generated heavy sample as an internal reference, enabling SILAC-based quantification even for samples that cannot be directly labeled. Here, we combine DIA-SiS, leveraging the robust quantification of SILAC without the complexities associated with chemical labeling. We developed DIA-SiS and rigorously assessed its performance with mixed-species benchmark samples on bulk and single cell-like amount level. We demonstrate that DIA-SiS substantially improves proteome coverage and quantification compared to label-free approaches and reduces incorrectly quantified proteins. Additionally, DIA-SiS proves effective in analyzing proteins in low-input formalin-fixed paraffin-embedded tissue sections. DIA-SiS combines the precision of stable isotope-based quantification with the simplicity of label-free sample preparation, facilitating simple, accurate, and comprehensive proteome profiling.

## Highlights

• Combining DIA with Spike-in SILAC for simple, accurate, and comprehensive proteomics.
• Evaluated by rigorous two-species benchmarking against label-free methods.
• Improved proteome coverage and quantification for bulk and single cell-like amounts.
• DIA-SiS is effective for low-input FFPE samples where metabolic labelling is impossible.
• Potential applications: low-input, single cell, clinical, high throughput.

## Graphical Abstract

**Figure:**
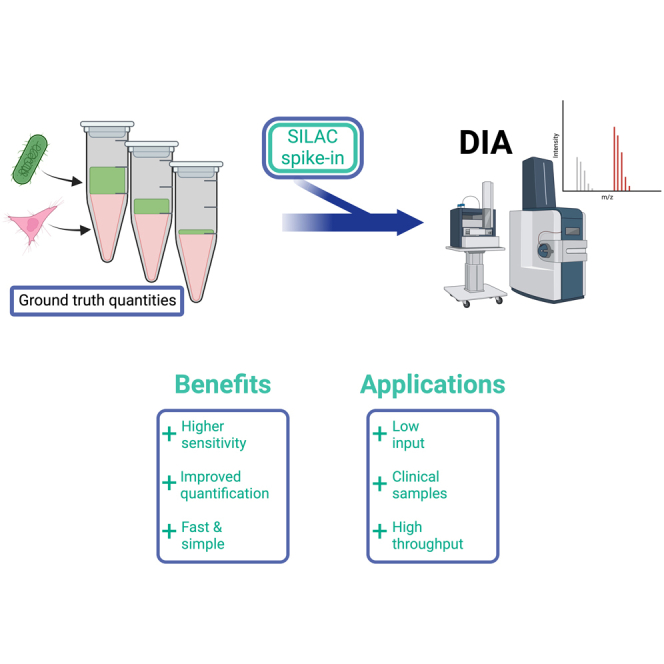

## In Brief

Typical bottom-up LC-MS-based proteomics experiments strive to measure as many proteins as possible both accurately and reproducibly. This can be achieved by metabolic labeling of proteins withSILAC. When SILACis used as a spike-in, it can be readily added to an unlabeled sample. Many researchers changed the way they operate the mass spectrometry to collect data from DDA to DIA. With rigorous mixed-species benchmarking experiments we demonstrate that DIA-SiS can improve coverage and quantification for bulk and single cell-like amounts.

In mass spectrometry-based proteomics, data-independent acquisition (DIA) is gaining popularity over data-dependent acquisition (DDA) for its higher throughput and fewer missing values (1, 2, 3, 4, 5, 6). Quantification in DIA typically uses label-free methods (7, 8, 9). However, since stable isotope-based quantification methods offer superior quantification in DDA (10), efforts have been made to extend those techniques to DIA. Chemical stable isotope labeling methods such as mass differential tags for relative and absolute quantification and dimethyl labeling have been successfully combined with DIA (11, 12). However, chemical labeling requires optimization to achieve high efficiency, and the additional *in vitro* steps complicate sample preparation and increase variability. Stable isotope labeling by amino acids in cell culture (SILAC) achieves high labeling efficiency through metabolic incorporation of heavy labels into proteins *in vivo* (13). This simplifies sample preparation since no extra chemical labeling steps are required. Moreover, combining differentially labeled samples early during sample preparation eliminates any variation that might result from sample processing. Due to these advantages, SILAC has become a popular method for functional proteomics, especially in combination with DDA (14, 15). As a metabolic labeling method, SILAC furthermore enables quantification of protein synthesis and degradation (16, 17, 18, 19). Recent studies successfully combined SILAC with DIA to study protein turnover (20, 21, 22, 23).

The primary limitation of SILAC is the need for metabolic incorporation of stable isotope-labeled amino acids, rendering it unsuitable for direct application in clinical scenarios (*e.g.*, human tissues) as well as certain high-throughput biological experiments. To address this issue, internal standard or “spike-in” methods have been devised (24, 25). These methods use SILAC to produce a heavy reference sample, which is subsequently added to each unlabeled (light) sample. The ratios of light to heavy proteins are determined in each sample. The uniform heavy reference across all samples serves as an internal standard, enabling relative quantification of light proteins across samples by computing the ratio of these ratios. In this way, spike-in SILAC (SiS) allows SILAC-based quantification for samples that cannot be directly labeled. A major advantage of SiS is the decoupling of labeling from sample preparation. Once the heavy spike-in reference is prepared and added, further processing requires no additional labeling, streamlining the workflow. Hence, SiS combines the accuracy of stable isotope-based quantification with the simplicity of label-free sample preparation. In combination with DDA, this methodology has been successfully implemented in both preclinical and clinical studies (26, 27, 28, 29, 30).

We reasoned that combining DIA with spike-in SILAC (DIA-SiS) would be a simple technique for sensitive, accurate, and precise quantification on a proteome-wide scale. First, DIA-SiS should provide better quantification than label-free approaches. Second, the addition of the heavy spike-in reference could facilitate detection of low abundant proteins. Third, the method is simple since it does not require additional chemical labeling steps. Here, we developed and rigorously benchmarked DIA-SiS on a mixed species dataset on bulk and single cell-like amounts with ground truth relative protein abundances. Our results show that DIA-SiS provides better quantification and improves the coverage, especially of low abundant proteins. In this way, DIA-SiS detects differentially abundant proteins with greater sensitivity and specificity. Additionally, and inspired by the super-SILAC approach (31), DIA-SiS can boost protein detection in low input formalin-fixed paraffin-embedded (FFPE) tissue sections to which a heavy multicell line super-spike-in mix was added. In summary, these data show that DIA-SiS significantly improves proteome coverage and quantification compared to label-free approaches.

## Experimental Procedures

### Sample Preparation

#### Two-species Benchmarking Experiment

The *Escherichia coli* strain AT713, which cannot synthesize lysine and arginine, was grown in a medium containing 0.5% (w/v) D-(+)-glucose (Sigma-Aldrich), 1.3% (w/v) M9 salts (BD), 1 mM MgSO4 (Merck), 377 μM thiamine (Sigma-Aldrich), 300 μg/ml L-arginine (Sigma-Aldrich) and L-lysine (Sigma-Aldrich), plus 150 μg/ml of the 18 other natural amino acids (Sigma-Aldrich). For heavy or light SILAC labeling, Arg10 (^13^C_6_,^15^N_4_) and Lys8(^13^C_6_,^15^N_2_) or Arg0 and Lys0 (Silantes) were used, respectively. To culture the bacteria, the glycerol stock was streaked onto 1.2% (w/v) lysogeny broth (LB, Sigma-Aldrich) agar plates and grown overnight. Then, single colonies were selected for overnight preculture in the defined SILAC light or heavy media. The precultures were then used to inoculate overnight batch cultures, again using the SILAC media. The colonies that were selected for this were tested for arginine and lysine auxotrophy on SILAC medium agar (1.2% w/v) plates supplemented with (1) nothing, (2) 300 μg/ml arginine, (3) 300 μg/ml lysine, and (4) 300 μg/ml arginine and lysine. Growth was only observed in condition 4.

The bacterial cells were harvested by pelleting (1500 relative centrifugal force (RCF), 4 °C, 10 m), then washed with ice-cold PBS (phosphate buffered saline, pH 7.4, Thermo Fisher Scientific). The lysis buffer (1% sodium deoxycholateSDC (Sigma-Aldrich), 150 mM NaCl (Roth), 100 mM Tris (Roth) pH 8, 1 Roche cOmplete protease inhibitor tablet per 10 ml) was added to the pellet to a target protein concentration of 1 mg/ml, vortexed and incubated for 10 m at 96 °C in a thermoblock. The mixture was frozen at −72 °C in a EtOH/dry ice slush and thawed at 30 °C, shaking at 1000 rpm in a thermoblock. The freeze-thaw cycle was repeated three times in total. The DNA was digested by incubation with benzonase (Sigma-Aldrich, 25 U per expected 1 mg of protein) for 30 min at room temperature (RT). Finally, the lysate was cleared for 15 m at 10,000 RCF and 4 °C. The protein concentration was determined by a bicinchoninic acid (BCA) assay (Pierce BCA Kit, Thermo Fisher Scientific). The disulfide bridges in the protein extract were reduced in 10 mM dithiothreitol (DTT, Sigma-Aldrich) for 1 h at 37 °C and shaking at 1000 rpm in a thermoblock. The free thiols were then alkylated by adding chloroacetamide (CAA, Sigma-Aldrich) to a concentration of 20 mM and incubating for 45 m at RT in the dark (1000 rpm, thermoblock) and the reaction subsequently quenched by increasing DTT to 50 mM. The sample cleanup and digestion into peptides was performed by protein aggregation capture on magnetic beads, based on (32). Briefly, 15:1 (w/w) magnetic beads (Cytiva SpeedBeads 45152105050250 and 65152105050250) to expected protein amount were added to the samples. Then, acetonitrile (ACN) concentration was increased to 75%. The beads with the proteins bound to them were then washed three times with 80% EtOH (CHEMSOLUTE) and then digested overnight at 37 °C and shaking at 1000 rpm (thermoblock) with trypsin (Sequencing Grade Modified Trypsin V5113, Promega) and LysC (Lysyl Endopeptidase 129–02541, Wako Chemicals) in a 1:50 protease to substrate ratio in ammonium bicarbonate buffer (Sigma-Aldrich, 50 mM). The next day, the peptides were lyophilized in a vacuum concentrator and resolved in solvent A (3% (v/v) ACN (CHEMSOLUTE) and 0.1% (v/v) formic acid (Fluka).

Human HL-60 cells were grown at 37 °C and 5% CO_2_ in T75 flasks. The cells were cultured at a density of 0.5 to 1.5 million cells per mL and pelleted at 300 RCF for 5 min for passaging. The unlabeled (light) cells were grown in RPMI Medium 1640 (Gibco, Thermo Fisher Scientific) supplemented with 10% fetal bovine serum (FBS, PAN-Biotech) and nonessential amino acids, sodium pyruvate as well as L-alanyl-L-glutamine (all Gibco, Thermo Fisher Scientific, according to the manufacturer's instructions). For heavy SILAC labeling, the cells were grown for 6 to 8 passages in SILAC RPMI Medium (PAN-Biotech, P04–02504) which lacks lysine and arginine. It was supplemented with heavy lysine and arginine (Lys8(^13^C_6_,^15^N_2_) and Arg10 (^13^C_6_,^15^N_4_), Silantes), 10% dialyzed FBS (PAN-Biotech), L-alanyl-L-glutamine (Gibco, Thermo Fisher Scientific), nonessential amino acids (Gibco, Thermo Fisher Scientific) and sodium pyruvate (Gibco, Thermo Fisher Scientific). Both labeled and unlabeled cells were harvested by pelleting for 5 min at 500 RCF and washed with ice-cold PBS (pH 7.4, Thermo Fisher Scientific).

For lysis, the cells were then resuspended by vortexing in a buffer composed of 1% (w/v) SDS (Roth), 5% (v/v) ACN(CHEMSOLUTE) in PBS (pH 7.4, Thermo Fisher Scientific) to a target concentration of 1 mg/ml. They were incubated for 10 min at 96 °C and subsequently sonicated in a Bioruptor plus ultrasonicator for ten cycles (30 s on/30 s off, 4 °C) on the high intensity sonication setting. Then, the lysates were cleared by centrifugation (15 m, 10,000 RCF, 4 °C) and the protein concentration determined by a BCA assay (Pierce BCA Kit, Thermo Fisher Scientific). The human protein extracts were further processed to peptides in the same manner as described above for bacterial protein extracts.

**Fig. 1:**
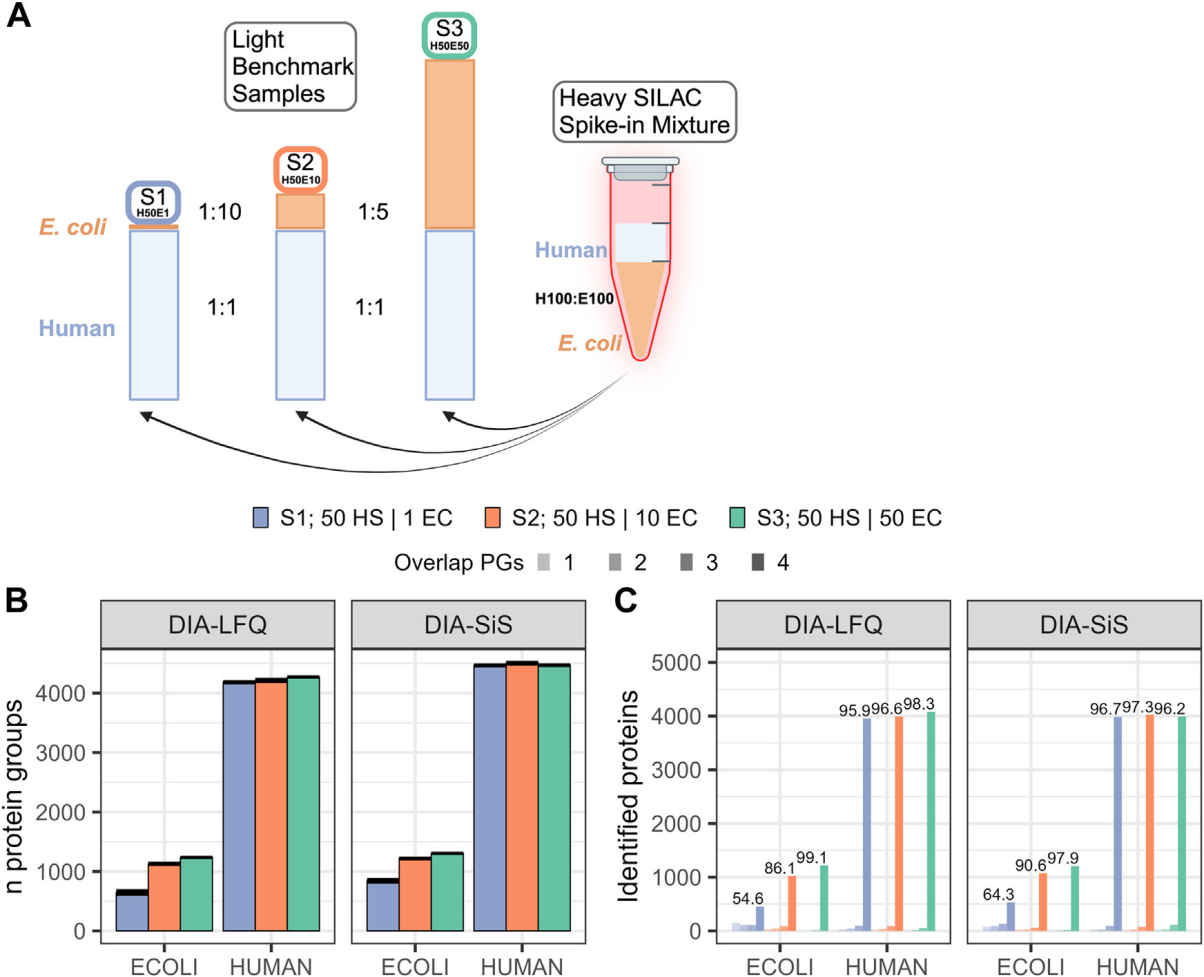
**Benchmark experiment: Design and protein coverage**. *A*, experimental design of the mixed species benchmark, amounts in ng. *B*, mean number of identified *Escherichia coli* and human HL-60 proteins over all four technical replicates per dilution step sample (standard deviation is indicated by *whiskers*). The different colors correspond to different *E. coli* dilutions. *C*, number of proteins identified in one, two, three, or all four replicates (increasing opacity). Numbers indicate the percentage of human or *E. coli* protein groups detected across all four replicates.

The peptides were then mixed according to Figure 1*A* and the injection amounts are indicated in the cartoon. For the low input samples (Fig. 3, *D*–*G*), 13.4 ng light *Homo sapiens* peptides with either 13.4 ng light *E. coli* peptides or no light *E. coli* peptides, plus as a spike-in 66.8 ng heavy *H. sapiens* peptides with 6.7 ng heavy *E. coli* peptides were injected. For the very low input samples (single cell-like amounts), the samples described in Figure 1*A* were diluted in low binding plasticware (Eppendorf twin.tec PCR plate LoBind) to avoid peptide loss at high dilutions. For the lowest *E. coli* dilution, 300 pg human and 300 pg *E. coli* peptides were injected with the heavy-spike in an excess of 2×, 5×, or 20× (for each species, excess with respect to the lowest *E. coli* dilution; supplemental Fig. S9)—or without any spike-in, in the case of label-free.

#### Super-DIA-SiS

For the super-SILAC spike-in mix, six different human papillomavirus negative head and neck squamous cell carcinoma cell lines (A-253 (salivary gland), Cal-33 (tongue), FaDu (pharynx), UT-SCC-14 (tongue), SCC-25 (tongue), and UPCI-SCC-026 (tongue)) were grown at 37 °C and 5% CO_2_ in 10 cm and 15 cm (last two passages) dishes. After thawing, cells were kept on Dulbecco's modified Eagle's medium (Gibco, Thermo Fisher Scientific) supplemented with 10% FBS (PAN-Biotech) for two to three passages until they recovered. After recovery, the medium was exchanged to SILAC Dulbecco's modified Eagle's medium (Pan-Biotech, P04–02501) supplemented with 10% dialyzed FBS (Pan-Biotech), L-alanyl-L-glutamine (Gibco, Thermo Fisher Scientific, according to the manufacturer's instructions) and heavy lysine and arginine (Lys8 and Arg10, Silantes). In order to receive maximal labeling efficacy, cells were split 1:4 at 70 to 80% confluency six times over the course of five to 6 weeks. At each passage, cells were washed with PBS (pH 7.4, Thermo Fisher Scientific) and incubated with 2 ml 0.05% Trypsin EDTA (Gibco, Thermo Fisher Scientific) for 10 to 15 min to detach the cells. Trypsin was blocked by adding 2 ml of medium. To avoid light lysine and arginine contamination from Trypsin EDTA, cells were pelleted for 5 min at 300 RCF and resuspended in the new medium.

For harvesting, the dishes were washed three times with ice-cold PBS (pH 7.4, Thermo Fisher Scientific) and transferred to microcentrifuge tubes using cell scrapers. The cell suspension was centrifuged at 16,000 RCF for 15 min at 4 °C. Cell pellets were stored in −80 °C freezer until further usage. For the lysis, the pellets were suspended in lysis buffer (50 mM Tris (Roth) pH 8, 1% SDS (w/v) (Roth), 150 mM NaCl (Roth), 1 Protease Inhibitor tablet (Roche cOmplete) per 10 ml) and boiled for 5 min at 95 °C. Lysates were ultra sonicated (Bioruptor Plus) for ten cycles (30 s on/30 s off) on high intensity sonication setting and centrifuged at 16,000 RCF for 15 min at RT to pellet the cell debris. Protein concentration of each lysate was measured *via* BCA (Pierce BCA Kit, Thermo Fisher Scientific).

Head and neck squamous cell carcinoma (HNSCC) FFPE samples were kindly provided by Dr Ingeborg Tinhofer-Keilholz (Laboratory for Translational Radiooncology, Dept. of Radiation Oncology and Radiotherapy, Charité Universitätsmedizin Berlin). Deparaffinization was done following a partially modified protocol from the High Pure FFPET DNA Isolation Kit (Roche). Briefly, FFPE slices were bathed in xylene (10 min at RT) and twice in fresh absolute ethanol (10 min at RT) and scraped into a tube. Samples were then centrifuged to pellet the tissues at 16,000 RCF for 2 min at RT. Pellets were washed by adding 70% ethanol, 5 seconds of vortexing, and pelleting the tissue again at 16,000 RCF for 4 min at RT. Ethanol was removed, ultrapure water was added, and the solution centrifuged again at 16,000*g* for 10 min at RT. The pellets were stored at −80 °C until further use. For the lysis a lysis buffer (4% (w/v) SDS (Roth), 25 mM Tris (Roth), 2.5 mM DTT (Sigma-Aldrich)) was added and the samples were incubated at 900 rpm for 2 h at 95 °C. The samples were then sonicated with a Covaris LE220Rsc at 250 W for 5 min. The samples were subsequently transferred back into microcentrifuge tubes and incubated at 900 rpm for 90 min at 95 °C. Pellet and suspension were then separated at 12,700 rpm for 30 min. The protein concentration was measured *via* BCA (Pierce BCA Kit, Thermo Fisher Scientific).

The FFPEs were either processed alone (300 ng and 50 ng samples) or together with the spike-in (50 ng + spike-in). To this end, equal amounts of the six different heavy labeled cell lysates were mixed to generate a super-SILAC spike-in mixture. This mixture was added to the FFPE extracts in such a way that the protein amount of the spike-in was five times the amount of FFPE proteins. The samples were processed (reduction, alkylation, and single-pot solid-phase-enhanced sample preparation) in three batches in a semiautomated manner in a 96 well plate using an Opentrons OT-2 (Opentrons Labworks Inc). Briefly, samples were reduced by incubating with 10 mM DTT (Sigma-Aldrich) for 30 min at RT shaking and alkylated with 20 mM iodoacetamide (Sigma-Aldrich) for 30 min at RT shaking. Alkylation was quenched by adding DTT (Sigma-Aldrich) to a final concentration of 50 mM and incubation for 15 min at RT shaking. The protein cleanup and digestion steps followed the same protocol as described for the benchmarks besides using a 20:1 bead to protein ratio. After digestion, peptides were further cleaned using a G5563A Bravo platform (Agilent Technologies). Briefly, the syringes of the robot’s pipetting head were washed with a washing buffer (50% ACN (CHEMSOLUTE) and 0.1% trifluoroacetic acid (Sigma-Aldrich)) to flush potential contaminants. The same buffer was used to condition the resin of an AssayMAP C18 cartridge rack. A solution of 0.1% trifluoroacetic acid (Sigma-Aldrich) was run through the cartridge to equilibrate them. Afterward, the samples were loaded onto the resin. Syringes and cartridge were washed with the washing buffer before eluting the samples in a fresh 96-well plate using the washing buffer. Afterward, the peptides were lyophilized in a vacuum concentrator and resolved in solvent A (3% (v/v) ACN (CHEMSOLUTE) and 0.1% (v/v) formic acid (Fluka)).

#### Liquid Chromatography and Mass Spectrometry

**Fig. 2:**
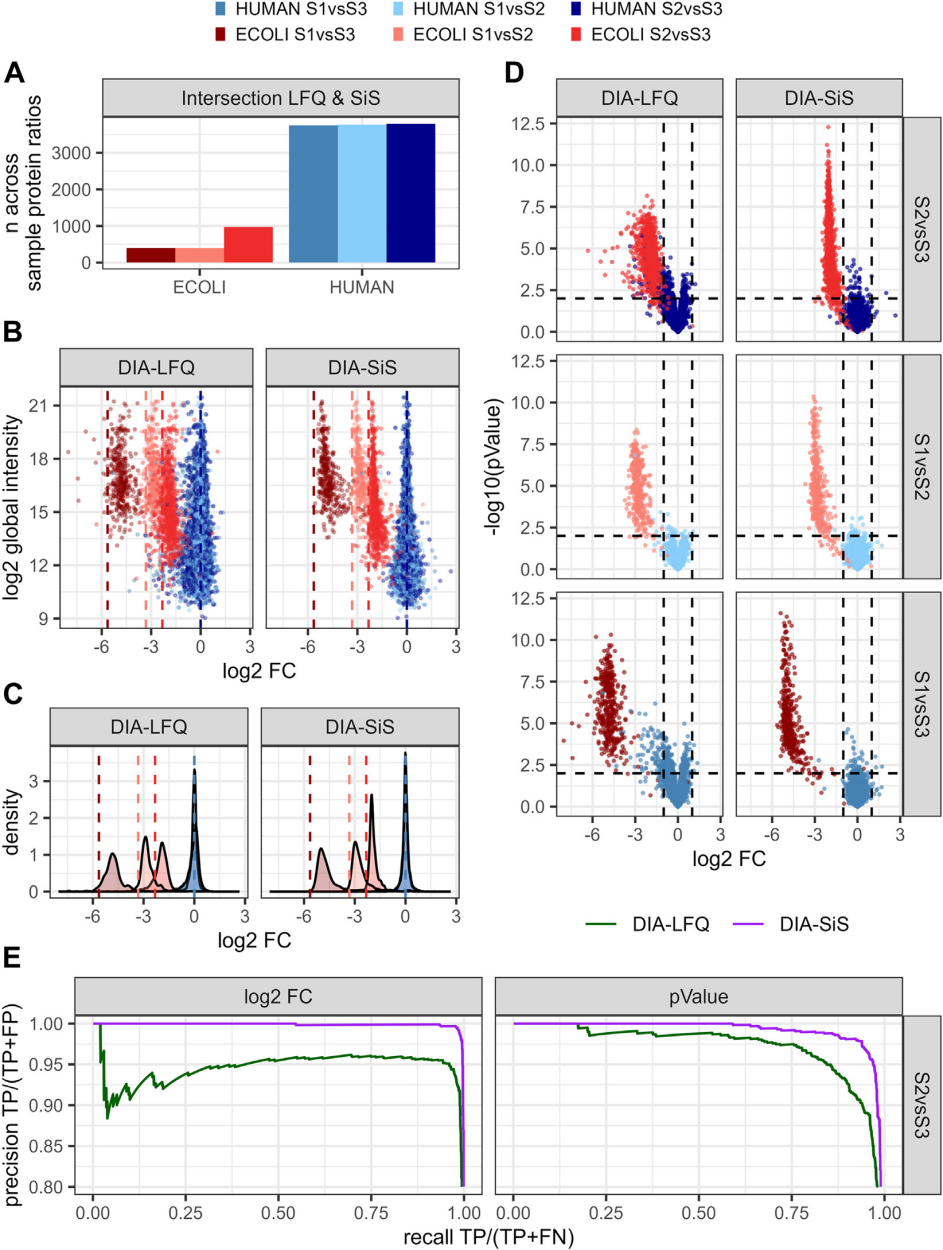
**DIA-SiS allows reliable across-sample quantification**. Only proteins with both LFQ and SILAC ratios and no missing values across all replicates are shown. *A*, the bars indicate the number of across-sample protein ratios quantified with both LFQ and DIA-SiS. *B*, both DIA-LFQ and DIA-SiS capture the expected across-sample ratios, with a clearer intensity-dependent precision for DIA-SiS. The global protein group abundance is plotted against the mean across-sample protein ratios. *Dashed lines* indicate expected ratios. *C*, density plots corresponding to (*B*). *D*, DIA-SiS improves the detection of differentially abundant proteins. The volcano plots show the log2FCs on the x axis and the -log10 *p*-values on the y axis. The *dashed lines* indicate cutoffs (*p*-value = 0.01, absolute log2FC = 1), *blue*: human proteins, *red*: *Escherichia coli* proteins. *E*, precision-recall curves based on (*D*) of S2 *versus* S3 for log2FCs (*left*) and *p*-values (*right*) for DIA-LFQ (*green*) and DIA-SiS (*purple*). DIA, data-independent acquisition; DIA-SiS, DIA with spike-in SILAC; FC, fold change; LFQ, label-free quantification; SILAC, stable isotope labeling by amino acids in cell culture.

**Fig. 3:**
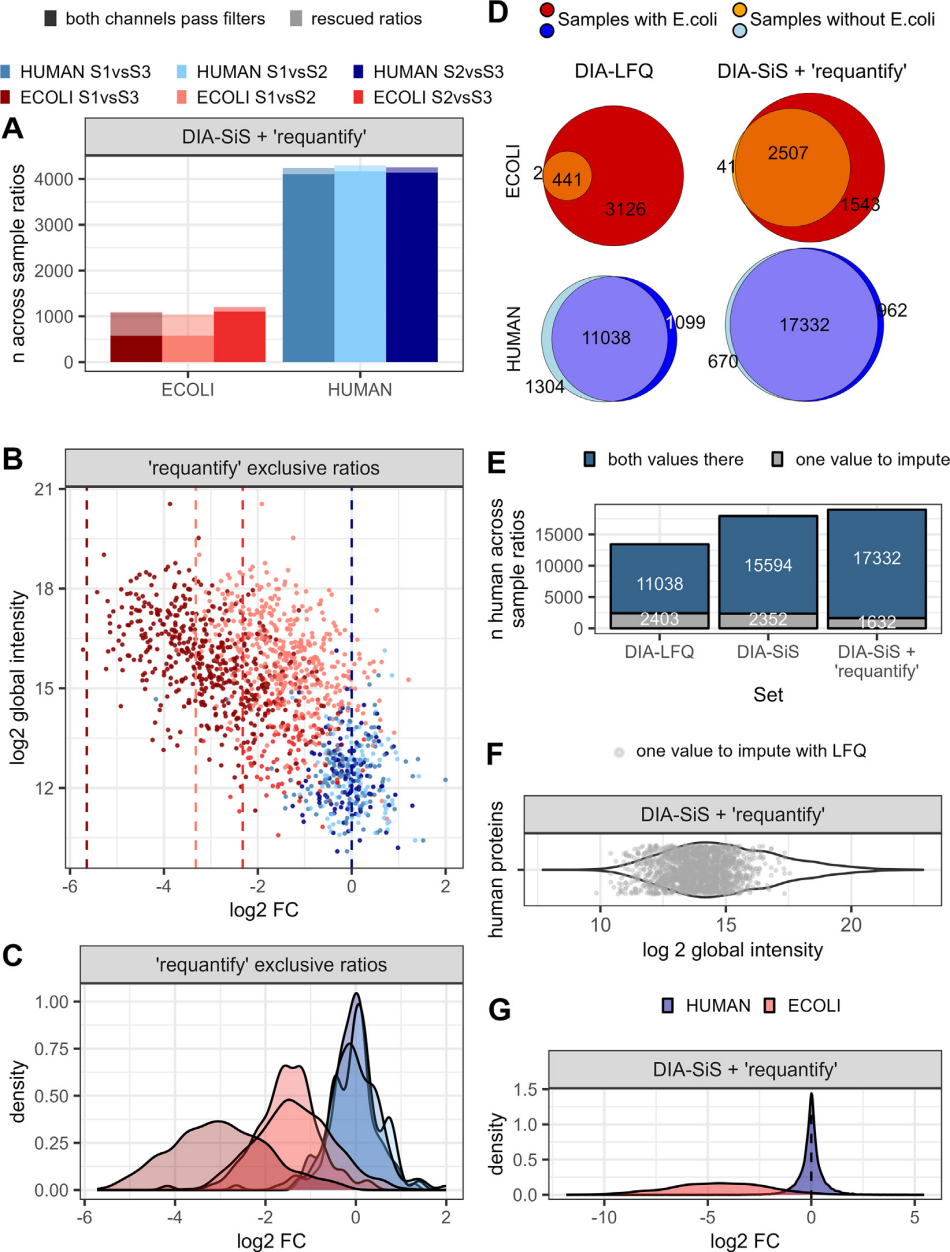
**"Requantify" further reduces missing values in DIA-SiS**. *A*, the number of proteins quantified across samples can be increased by only requiring confident identification of heavy channel precursors (“requantify” option). Mean number of across sample ratios (without missing values) using DIA-SiS, requiring the light as well as heavy channel to pass filters (higher opacity) and additional ratios obtained when using "requantify" (*lower opacity*). *B*, “requantified“ DIA-SiS ratios still capture the correct trend. The log global abundance is plotted against the mean log2FCs of protein groups (*blue*: human proteins, *red*: *Escherichia coli* proteins). *C*, density plots corresponding to (*B*); *D*–*G*, comparison of a sample containing human and *E. coli* proteins to sample without *E. coli* shows that DIA-SiS reduces missing values, especially with "requantify" enabled. *D*, cumulative Venn diagrams of *E. coli* (*upper*, *red*/*orange*) and human (*lower*, *blue*) proteins quantified in four technical replicates. *E*, DIA-SiS also reduces missing values for human proteins, although they were equally abundant in both samples. Cumulative bar charts for the human proteins illustrated in (*D*) indicate the number of ratios that could be computed between samples. *F*, rescued human proteins cover a broad abundance range. The cumulative distribution shows the log2 global intensity of those proteins that are missing in LFQ but could be rescued with DIA-SiS and "requantify". The *violin* indicates the intensity of the entirety of proteins found with DIA-SiS and "requantify". *G*, across-sample ratios obtained using SILAC + "requantify" correctly capture the global trend (*blue*: human, *red*: *E. coli*). SILAC, stable isotope labeling by amino acids in cell culture; DIA, data-independent acquisition; DIA-SiS, DIA with spike-in SILAC; LFQ, label-free quantification.

DIA measurements of the main benchmark (Fig. 1, Fig. 2, Fig. 3), the human only benchmark (supplemental Fig. S3) and our Super-DIA-SiS (Fig. 5) approach were performed on a trapped ion mobility mass spectrometry time-of-flight (timsTOF) Pro 2 (Bruker Daltonics) attached to an EASY-nLC 1200 (Thermo Fisher Scientific) in DIA-parallel accumulation–serial fragmentation (PASEF) mode using an in-house packed column (20 cm long, 75 μm diameter, 1.9-μm ReproSil-Pur C18-AQ silica beads, Dr Maisch) heated to 50 °C connected to a nanoelectrospray ion source (CaptiveSpray; Bruker Daltonics) with a spray voltage of 1600 V. The peptides were separated at an increasing percentage of solvent B using a ∼30 min active gradient at a constant flow rate of 250 nl/min: 0 min 2%; 1 min 7%; 20 min 20%; 29 min 30%; 32 min 60%; 33 min 90%. Solvent A consisted of 3% ACN (LC-MS grade, CHEMSOLUTE) and 0.1% formic acid (Fluka) in H2O (LC-MS grade, CHEMSOLUTE) and Solvent B of 90% ACN (LC-MS grade, CHEMSOLUTE) and 0.1% formic acid (Fluka) in H2O (LC-MS grade, CHEMSOLUTE). The acquisition was operated in DIA-PASEF (33) mode with the mass range set to 400 to 1200 m/z, mobility range set to 0.60 to 1.60 V∗s/cm2, a ramp time of 100 ms and an accumulation time of 100 ms with an estimated cycle time of 1.80 s and a collision energy with a linear ramp from 20.00 eV at 0.60 V∗s/cm2 to 59.00 eV at 1.60 V∗s/cm2. The DIA-PASEF scheme covers the mass range with 2 m/z windows divided into 16 TIMS ramps of 25 m/z (“DIA-PASEF long gradient”, as provided by Bruker Daltonics).

The single cell-like amounts experiment was measured on a timsTOF SCP (Bruker Daltonics) with the CaptiveSpray source at 1500 V in a very similar setup as described above. A ∼ 14 min active gradient with an increasing percentage of solvent B was used: 0 min 2% 400 nl/min; 0.6 min, 7% 400 nl/min; 1.5 min 10% 400 nl/min; 10 min 20% 250 nl/min; 14 min 33% 250 nl/min; 15 min 60% 250 nl/min; 16 min 90% 250 nl/min. Operated in DIA-PASEF mode as well, the mass range extended from 400 to 1000 m/z and the ion mobility range from 0.64 to 1.37 V∗s/cm2 with a ramp as well as accumulation time of 100 ms and an estimated cycle time of 0.96 s and a collision energy with a linear ramp from 20.00 eV at 0.60 V∗s/cm2 to 59.00 eV at 1.60 V∗s/cm2. The DIA-PASEF acquisition scheme covered the mass range with three m/z windows divided into eight TIMS ramps of 25 m/z.

To check the SILAC labeling efficiency, samples were measured in DDA mode on an Orbitrap Exploris 480 (Thermo Fisher Scientific) coupled to a Vanquish Neo UHPLC-System (Super-DIA-SiS; Thermo Fisher Scientific) or an EASY-nLC 1200 (benchmarks; Thermo Fisher Scientific) with an electrospray ionization source (Thermo Fisher Scientific) using same in-house packed columns as described above for reverse phase separation. Over a ∼30 min (Super-DIA-SiS) or ∼ 90 min (benchmarks) active gradient and a constant flow rate of 250 nl/min peptides were eluted (30 min active gradient: 0 min, 2%; 1 min, 7%; 20 min 20%; 29 min 30%; 32 min 60%; 33 min 90%; 90 min active gradient: 0 min, 2%; 1 min, 4%; 68 min 20%; 88 min 30%; 98 min 60%; and 99 min 90%). The elution solvent A and B composition was the same as for the DIA measurements. Standard mass spectrometry settings were used, which are briefly: (1) 30 min active gradient Top20: MS1: 60k Orbitrap resolution, 300% normalized automatic gain control (AGC) target, 10 ms maximum injection time and 350–1600 m/z scan range. For MS2, isolation width was 1.3 Da, 28% normalized higher-energy collisional dissociation energy, Orbitrap resolution 15k, 100% AGC target with 22 ms maximum injection time and 20 s dynamic exclusion with 10 ppm mass tolerance; (2) 90 min active gradient Top20: MS1: 60k Orbitrap resolution, 300% normalized AGC target, 10 ms maximum injection time, and 350 to 1600 m/z scan range. For MS2, isolation width was 1.3 Da, 28% normalized higher-energy collisional dissociation energy, Orbitrap resolution 15k, 100% AGC target with 22 ms maximum injection time, and 20 s dynamic exclusion with 10 ppm mass tolerance.

#### Labeling Efficiency

Raw files were analyzed with MaxQuant 1.6.7.0 (https://www.maxquant.org) with the Andromeda search engine against the respective UniProt FASTA database, UP000005640 (Human) and UP000000625 (*E. coli*), set to Trypsin/P and specific mode with contaminants FASTA from MaxQuant and Arg10 and Lys8 as heavy labels. The peptide spectrum match and protein false discovery rate was set to 0.01. Further search settings include the following: variable modifications: oxidation (M), acetylation (N-terminal); fixed modifications: carbamidomethylation (C); Max. missed cleavages: 2; Mass tolerance: MS1 first search 20 ppm, MS1 after calibration 7 ppm and MS2 20 ppm. The evidence files were filtered for peptides containing one arginine or lysine and sorted after their intensity. Some of the highest intensity precursors with low posterior error probability were visualized using Thermo Xcalibur 4.6.67.17 Qual Browser (Thermo Fisher Scientific, https://www.thermofisher.com/xcalibur) and Inkscape 1.1 (https://inkscape.org/).

### Experimental Design and Statistical Rationale

Since we did not aim to investigate biological differences, we measured all experiments in technical replicates. These technical replicates are required to assess the reproducibility of identifications as well as the precision of the quantifications. Spike-in amounts for the DIA-SiS samples were selected based on our prior experiments (data partially discussed in Fig. 3, *D* and *E*) and theoretical considerations (*i.e.* using more spike-in than sample). In total, we provide four different benchmark datasets: The mixed-species dataset (maximum 50 ng light input per species) with 2× spike-in excess, the mixed-species single cell-like amount dataset (maximum 300 pg light input per species) and a human only dataset to assess upper input limitations. We also provide data from another mixed-species dataset that generally uses less spike-in and was used to showcase the usage of our method if proteins (*E. coli*) are not there (Fig. 3, *D* and *E*). While we only use it to present this scenario, the whole data set is published as well. In addition to all the benchmark datasets, we provide proteomic data of 16 FFPE samples (two of them are presented in this article) with and without spike-in. For all the mixed-species benchmarking experiments, the dilutions were measured in technical quadruplicates, while the two FFPE samples were measured in technical triplicates and the human only benchmark in technical duplicates.

### Data Processing

Raw files were processed with DIA-NN v 1.8.1 (https://github.com/vdemichev/DiaNN). In order to increase the analysis speed, we generated specific refined libraries for the benchmark and the Super-DIA-SiS experiments. For the mixed species benchmarks, FASTA files for *E. coli* (UP000000625, accessed 2023/01/25), *H. sapiens* (UP000005640, accessed 2023/01/25) and contaminants (Universal Contaminant Protein FASTA from (34)) were concatenated and used for the generation of a predicted library using the following DIA-NN settings: --fasta-search --min-fr-mz 200 --max-fr-mz 1800 --met-excision --cut K∗,R∗ --missed-cleavages 1 --min-pep-len 7 --max-pep-len 30 --min-pr-mz 300 --max-pr-mz 1800 --min-pr-charge 1 --max-pr-charge 4 --unimod4 --reanalyze --relaxed-prot-inf --smart-profiling --peak-center --no-ifs-removal.

The resulting predicted library was then used as the basis for library refinement. For the benchmarks, the refinement was based on quadruplicates of the label-free samples (using the lowest dilution, *i.e.*, the samples with the largest amount of *E. coli* present). For library refinement, DIA-NN settings were left at default besides changing the library generation setting to “IDs, RT and IM profiling”.

For the generation of the predicted library for our Super-DIA-SiS approach, the same *H. sapiens* and contaminants FASTA files as used in the two-species benchmark experiment were combined and used to predict the library. This library was refined using the first technical replicate per FFPE sample of the high input label free measurements (300 ng).

For the very-low input samples (single cell-like amounts) the same predicted library was used but refined with quintuplicate 20 ng (10 ng *E coli* plus 10 ng human) label-free samples. All samples then have been analyzed using this refined library and using the same DIA-NN settings as for the other benchmarks.

For supplemental Fig. S8, raw data were searched with Spectronaut (version 18.5.231110, https://biognosys.com/software/spectronaut/) with default settings. For library generation, label-free quadruplicate measurements of the lowest *E. coli* dilution were used, similar to what was described above for DIA-NN. For labeled data, in-silico generation of missing channels was enabled and the workflow set to "label". This was then used to search the raw data with default settings, except for setting the "Multi-channel Workflow Definition" to "From Library Annotation" with the "Fallback Option" "Labeled". For label-free data, the library was based on the same files as for the labeled library, but without defining labels and otherwise retaining default settings. In all cases, reports were filtered for contaminants, FG.Charge >1, PG.QValue (Run-Wise) < 0.01, EG.QValue <0.01 and EG.IsDecoy = F. The PG.Quantity column was used to calculate across-sample ratios for both label-free quantification (LFQ) and DIA-SiS data and normalized as described for the DIA-NN benchmark output. Data analysis and most of the figures were done in R 4.2.2 (https://www.r-project.org/) in RStudio 2022.12.0 Build 353 (https://posit.co/download/rstudio-desktop/), additionally using packages *tidyverse* 2.0.0 (35), *RColorBrewer* 1.1.3 (https://cran.r-project.org/package=RColorBrewer), *ComplexUpset* 1.3.3 (http://doi.org/10.5281/zenodo.3700590, (36), *pROC* (37), *eulerr* 7.0.1 (https://cran.r-project.org/package=eulerr) and *ggpubr* 0.6.0 (https://cran.r-project.org/package=ggpubr). Experimental designs have been generated with BioRender (https://www.biorender.com/), and figures have been polished using Affinity Designer 2 2.3.0 (https://store.serif.com/de/update/windows/designer/2/).

#### The report.tsv Files from the DIA-NN Output Were Used for Further Data Analysis

LFQ reports were filtered for contaminants, Precursor.Charge >1, Lib.PG.Q.Value < 0.01 and Lib.Q.Value < 0.01. The column PG.MaxLFQ was used as quantitative output for each protein group (PG). SILAC reports were filtered for contaminants and Precursor.Charge >1. Additionally, Global.PG.Q.Value < 0.01 and Channel.Q.Value < 0.03 were used as filters for (a) both, the light and heavy channel (basic filtering) and (b) only the heavy channel ("requantify"). For "requantify", if only the heavy channel passed q value filtering, the light precursor was allowed to pass as well. For LFQ and SILAC data of the main benchmark (Fig. 1, Fig. 2, Fig. 3), the human only benchmark (supplemental Fig. S3) and the Super-DIA-SiS approach (Fig. 5), Ms1.Translated and Precursor.Translated columns were both required to contain at least one valid value and be > 0. For the timsTOF SCP benchmark, any of them was required to pass the above filters due to many missing values and zeros in one of them. If both columns were available, both columns were used for the Precursor L/H calculation, if not, just the remaining column was used.

Precursor SILAC ratios were calculated by dividing the light “Ms1.Translated” or “Precursor.Translated” precursor intensities by the corresponding heavy partner. After log10 transformation, protein level L/H ratios were calculated by taking the median of all “Ms1.Translated” and “Precursor.Translated” L/H ratios for all precursors of a given protein. Light protein abundances have been calculated by multiplying (*i.e.*, adding, in log space) the protein L/H ratio with the global median heavy intensity of that protein. The global median heavy intensity was calculated by (1) summing up all precursor intensities (Ms1.Translated and Precursor.Translated) for each protein and (2) taking the median log10 of these summed intensities across all samples.

The mixed-species benchmark quantities were normalized to the human across-sample ratios and to dilution S3. Across-sample ratios were calculated for S1 *versus* S3 and S2 *versus* S3 per replicate by dividing protein L/H abundances (spike-in data) or LFQ (label-free data) values of two samples. These across-sample ratios were used to calculate the difference between the theoretical human (*i.e.* 1:1) and the observed median human log2FC (fold change, FC). The shift of S1 *versus* S3 was subtracted from S1, the shift of S2 *versus* S3 from S2. S3 was not shifted. These corrected abundances were then used to calculate the final log2FCs and *p*-values.

For the across-sample comparisons of log2FCs, proteins with missing values in any replicate were ignored.

For the human only benchmark data (supplemental Fig. S3), we only assessed identifications, not quantitative performance. Therefore, no normalization was performed.

In order to normalize the Super-DIA-SiS data, protein L/H ratios in one run were shifted by the median of all L/H protein ratios of that run before calculating the abundances. The LFQ FFPE data were already normalized by DIA-NN.

**Fig. 4:**
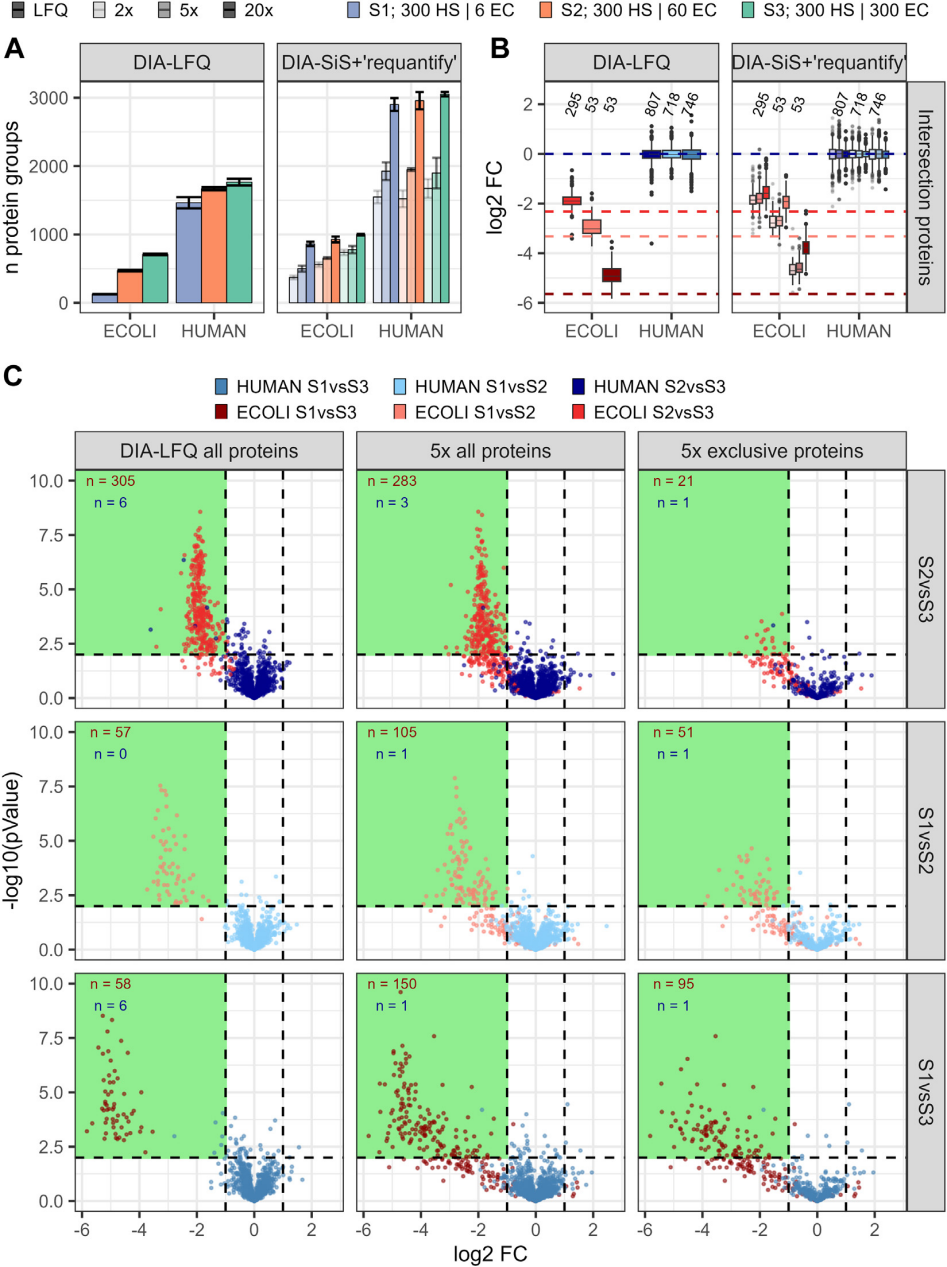
**DIA-SiS boosts IDs for single cell-like amounts**. For this analysis, the samples from Fig. 1 A were further diluted to single cell-like amounts (supplemental Fig. S9). The lowest dilution contains 300 pg human and 300 pg *E. coli*. The samples have been measured without spike-in (LFQ) or with 2×, 5×, and 20× the amount of spike-in as sample (increasing opacity). Across sample ratios have been calculated for proteins detected across all four replicates. *A*, number of proteins quantified with each approach. *B*, distribution of across-sample ratios (log2FC) of intersecting proteins showing the ratio compression with increasing spike-in amounts. Numbers indicate the number of proteins per comparison. *C*, volcano plots of all proteins found with LFQ and 5× SILAC as well as the 5× exclusive ones. Numbers indicate the number of *E. coli* (*red*) and human (*blue*) proteins with a log2FC <= −1 and a *p*-value <0.01 (within the *green rectangle*). DIA, data-independent acquisition; DIA-SiS, DIA with spike-in SILAC; LFQ, label-free quantification; SILAC, stable isotope labeling by amino acids in cell culture.

**Fig. 5:**
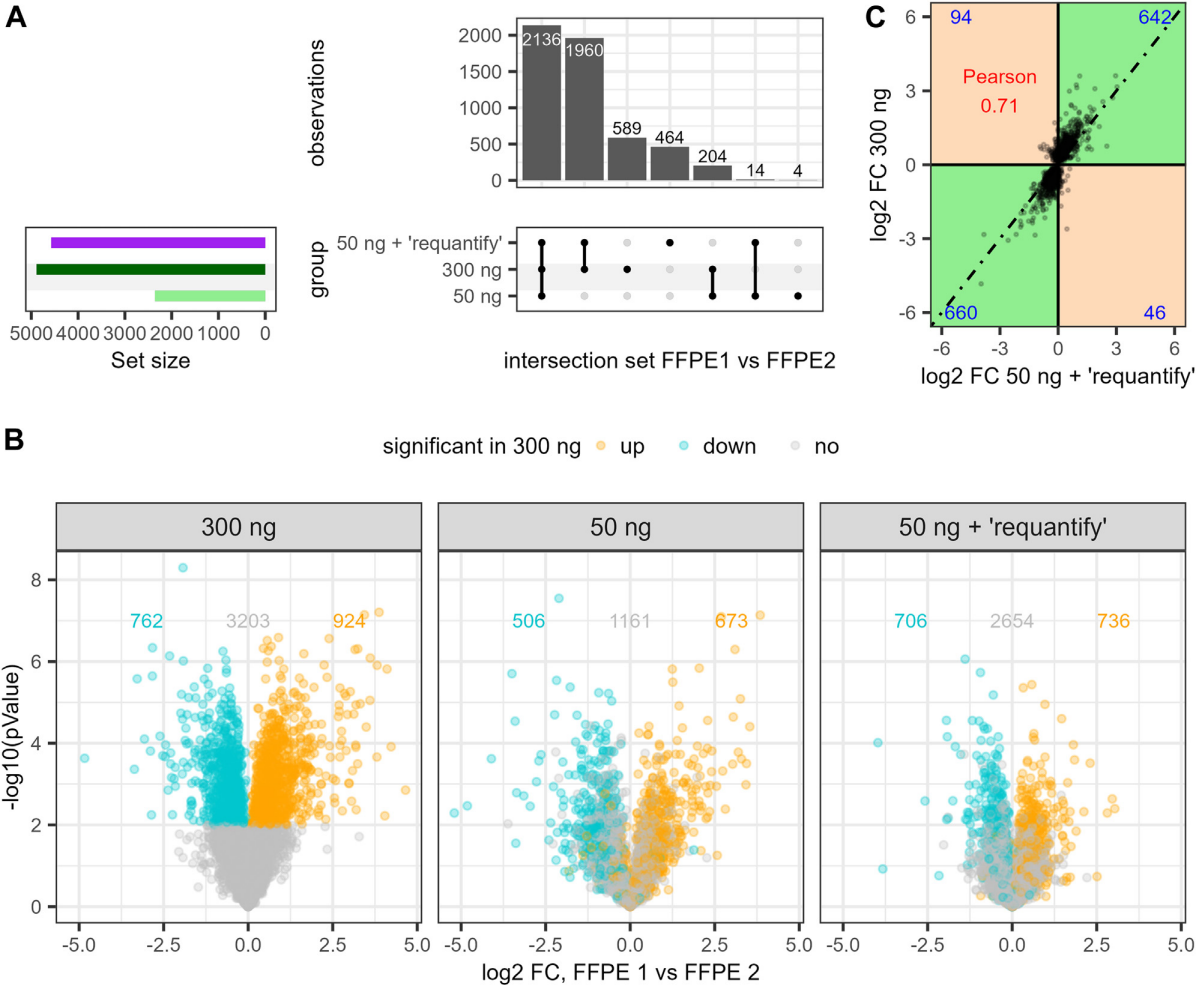
**Application of DIA-SiS (with “requantify”) to formalin-fixed paraffin-embedded (FFPE) head and neck squamous cell carcinoma (HNSCC) samples**. Two FFPE tissue samples (FFPE1 and FFPE2) were compared using different input amounts: 300 ng, 50 ng and 50 ng + 250 ng of the super-SILAC spike-in reference with “requantify” (50 ng + “requantify”). *A*, number of across-sample ratios obtained. Reducing the input from 300 to 50 ng reduces the number of proteins that can be quantified. Adding the spike-in recovers most of the proteins lost in the low input sample. *B*, volcano plots for differentially abundant proteins in the FFPE1 *versus* FFPE2 sample for the different input amounts. Significantly differentially abundant proteins (*turquoise* and *orange*) were defined based on the 300 ng input (*p*-value ≤ 0.01) and colored accordingly in the other samples. The number of proteins in each subset is indicated. *C*, the correlation of the log2FCs of the differentially abundant protein between the 300 ng input and the 50 ng + ref. input is high. The number of proteins in the quadrants is indicated. SILAC, stable isotope labeling by amino acids in cell culture; DIA, data-independent acquisition; DIA-SiS, DIA with spike-in SILAC; FFPE, formalin-fixed paraffin-embedded.

For the differential abundance analysis in Figures 2, 4, and 5, we performed two-sided t-tests without multiple testing correction. For Figures 2*D* and 4*C* proteins with a *p*-value <0.01 and an absolute log2FC > 1 was considered significantly differentially abundant while for Figure 5*B* only the *p*-value cutoff was used for the 300 ng samples. To generate the precision-recall curves (Fig. 2*E*), only proteins with negative fold changes were used.

For the comparison with directLFQ (7) in supplemental Fig. S5, the label-free report.tsv was q-value filtered as described above. The filtered report was processed by the directLFQ package (https://github.com/MannLabs/directlfq, version 0.2.19) using default settings. The resulting directLFQ values were normalized and analyzed together with and in the same manner as the DIA-NN LFQ and DIA-SiS data sets.

In order to enable a fair comparison between label-free FFPE data and DIA-SiS FFPE data, the proteins were reduced to proteins found in any of the spike-in samples. This reduces the proteins under consideration by ca. 7.5 to 8.2% (from 5500-6000 with 300 ng LFQ) and by ca. 6.3 to 10% (from 3000-4800 with 50 ng LFQ).

### Statement of Ethics

Study approval statement: This study protocol was centrally reviewed and approved by the LAGeSo Ethics Cie Berlin, based on the principles in the Declaration of Helsinki (approval number [ZS EK 10 310/09]). Consent to participate statement: Written informed consent was obtained from all individual participants included in the study.

## Results and Discussion

### DIA-SiS Improves Protein Coverage, Especially for Low Abundant Proteins

To establish DIA-SiS, we first generated samples for rigorous benchmarking (Fig. 1*A*). A popular method to generate such samples is to combine proteomes of different species in defined ratios (8, 9, 11). Those across-sample ratios constitute a ground truth and can therefore be used to assess the accuracy of a quantitative proteomics method. To this end, we mixed protein extracts from human HL-60 cells and *E. coli*, keeping the amounts of human proteins constant while diluting *E. coli* proteins down to a ratio of 1:50 (Fig. 1*A*). To obtain heavy SILAC spike-in samples that contain both heavy human and *E. coli* proteins, we labeled both human HL-60 and *E. coli* cells with heavy stable isotope-encoded lysine and arginine (Lys-8 and Arg-10). Since WT *E. coli* is prototrophic for all amino acids, we used the strain AT713 that is unable to synthesize lysine and arginine and has previously been used for SILAC labeling (38). We observed high labeling efficiencies for both human and *E. coli* and added both samples to obtain mixed-species samples with a heavy spike-in reference for either species (supplemental Fig. S1*A*). Those were then benchmarked against mixed-species samples with the same light proteome composition, but which lack the heavy spike-in. All samples were analyzed in quadruplicates on a Bruker timsTOF Pro2 instrument using 44 min gradients in DIA-PASEF mode (33). We injected the same amounts with respect to the light content of the samples for both the label-free (light only) and DIA-SiS (light + heavy spike-in) analyses for a direct comparison of both approaches.

We analyzed the data using DIA-NN, an open-access software that has been widely adopted for DIA proteomics (6, 9). For label free quantification (LFQ), we relied on the MaxLFQ algorithm as implemented in DIA-NN (8). To enable DIA-SiS-based quantification we developed a pipeline that takes the DIA-NN output and uses the heavy spike-in as an internal reference for across-sample quantification. Briefly, we first obtain light to heavy (L/H) ratios for every protein in each sample from the median of its corresponding L/H precursor ratios. Next, we derive a global heavy intensity for each protein by taking the median of the summed heavy precursor intensities per protein across samples. Finally, for every protein, we multiply this global heavy intensity with the sample-specific L/H ratios to obtain sample-specific protein intensities. We provide the analysis pipeline in R and python on github (DOI will be made available upon acceptance).

We compared the number of PGs identified with DIA-LFQ and DIA-SiS (Fig. 1*B*). For human proteins, the numbers were similar with a mild increase of about 10% in DIA-SiS. For *E. coli* proteins, on the other hand, we observed a marked increase with DIA-SiS, especially for the sample containing the lowest amounts of *E. coli* proteins. Here, DIA-SiS identified about 30% more PGs than LFQ on average (ca. 900 *versus* 700). This is expected since the “peak translation” feature of DIA-NN can facilitate the detection of lower abundant light precursors by the presence of more abundant heavy precursors (11). This is also seen at the level of precursors: Except for the samples with the highest amount of *E. coli* (S3), DIA-SiS consistently yields a slightly higher number of precursors per protein (supplemental Fig. S2). We also assessed the effect of different loading amounts on protein coverage using human only samples. To this end, we added twice the amount of heavy spike-in (DIA-SiS) or no heavy spike-in (DIA-LFQ) to different amounts of light samples (10–200 ng). DIA-SiS offers superior coverage for up to 50 ng for the 21 min gradient and up to 100 ng for the 44 min gradient (supplemental Fig. S3*A*). These trends are also reflected in the number of proteins detected across replicates (supplemental Fig. S3*B*) and the number of precursors detected per protein (supplemental Fig. S3*C*). Together, these data show that for sensitivity, DIA-SiS is especially beneficial for low sample amounts.

We also investigated the data completeness across replicates. We therefore asked how often a given protein group was identified across the quadruplicate measurements (Fig. 1*C*). The majority (>90%) of human proteins were consistently detected in all four replicates with either approach. For lower *E. coli* amounts, DIA-SiS increases the data completeness. We conclude that LFQ and DIA-SiS provide overall similar proteome coverage, with advantages of DIA-SiS for low abundant proteins.

### DIA-SiS Enables More Reliable Protein Quantification

Next, we compared the quantification of DIA-SiS and LFQ. To this end, we computed mean across-sample ratios for each protein across the four technical replicates per sample. For a fair comparison, we first looked at proteins identified in all four replicates in both LFQ and DIA-SiS (Fig. 2*A*). For this subset, we plotted global protein intensities against the observed log2FCs. Reassuringly, both LFQ and DIA-SiS yielded protein quantities that are overall consistent with the expected ratios (Fig. 2, *B* and *C* and supplemental Fig. S4*A*). Also, the variance of the ratios was lower for more abundant proteins with both approaches. However, a closer comparison of LFQ and DIA-SiS revealed an overall higher precision of DIA-SiS (that is, reduced spread of log2 ratios). We made similar observations using the directLFQ package (7) instead of the MaxLFQ algorithm implemented in DIA-NN (supplemental Fig. S5). Therefore, the improved performance of DIA-SiS is not due to any specific limitations of the LFQ algorithm used.

To investigate the practical implications of the differences in the quantitative performance of LFQ and DIA-SiS, we used Student's *t* test to identify proteins that are significantly changing across replicates and present the data as volcano plots (Fig. 2*D*). While again, both LFQ and DIA-SiS were able to distinguish human (not changing) and *E. coli* (changing) proteins based on their FCs, DIA-SiS yielded a better separation of the two protein populations. For example, the small number of human proteins that appeared to be differentially abundant between samples in the DIA-SiS analysis typically had large (that is, nonsignificant) *p*-values. In contrast, the LFQ analysis resulted in a larger number of human proteins that appeared as differentially abundant, some of them with disturbingly small *p*-values. At a *p*-value cutoff of 0.01 and a log2FC cutoff of −1 (dashed lines) in the S1 *versus* S3 comparison, LFQ misclassifies 109 human proteins as significantly differentially abundant, while DIA-SiS only misclassifies a single protein. These observations are further supported by precision recall curves (Fig. 2*E*). Strikingly, DIA-SiS leads to substantially fewer erroneous differential abundance as well as *p*-value calls, improving the specificity. Thus, for the subset of proteins covered by both methods, we conclude that DIA-SiS improves quantification, which enables substantially better identification of differentially abundant proteins.

In addition to the subset of proteins quantified by both LFQ and DIA-SiS, we also looked at proteins exclusively covered in the DIA-SiS analysis (140–190 *E. coli* and 350–400 human proteins, supplemental Fig. S6*A*). As expected, we found that the precision of these ratios was worse (supplemental Fig. S6, *B* and *C*), and the number of human proteins erroneously classified as differentially abundant was higher (supplemental Fig. S6*D*) in this subset than for the intersection. However, the vast majority of SILAC exclusive proteins were still correctly classified. Additionally, we looked at all the proteins detected with either LFQ or DIA-SiS (supplemental Figs. S4*B* and S5*B*) where we observe a similar effect. Of note, the improved quantification with DIA-SiS is not entirely a consequence of the increased coverage on precursor level. With the same number of precursors per protein, we still observe a slight quantitative advantage of DIA-SiS over DIA-LFQ (supplemental Fig. S7). In summary, we conclude that DIA-SiS markedly improves the reliability of protein quantification.

### Heavy Spike-In Based Requantification Further Improves the Sensitivity of DIA-SiS

The findings presented above indicate that DIA-SiS improves protein detection, especially for the lower abundant *E. coli* proteins. This observation is expected due to the "peak translation" feature of DIA-NN (11). However, we reasoned that this does not yet take full advantage of the spike-in's potential for pinpointing low-abundance peptides: Since light and heavy precursors have identical ion mobility and very similar liquid chromatography retention times, detecting the heavy reference implies comprehensive coverage in the LC-MS run where the light precursor is anticipated. Therefore, confidently detecting the heavy spike-in reduces the chance that the absence of a corresponding light precursor is caused by technical issues. Based on this idea and inspired by a similar feature in MaxQuant (39), we implemented a "requantify" functionality in our pipeline. In MaxQuant, when a precursor is exclusively detected in one SILAC state (light or heavy), the “requantify” algorithm checks the raw file for any evidence of the corresponding SILAC partner that might have escaped detection and uses this signal for quantification. Instead of inspecting the raw data, our implementation of “requantify” is based on the report.tsv output of DIA-NN. If “requantify” is enabled, only the heavy reference is required to pass our filtering criteria. Any corresponding light precursor signal is then accepted by default, and its intensity values are used for quantification. While the low intensity values rescued in this way are expected to be noisy, we reasoned that they might still allow us to capture the right tendency. Enabling the "requantify" feature nearly doubles the quantifiable number of *E. coli* proteins in the S1 *versus* S3 comparison (Fig. 3*A*). Although these requantified ratios exhibit lower precision and accuracy, they correctly capture the expected trends (Fig. 3, *B* and *C* and supplemental Fig. S4*B*, right). For instance, the majority of *E. coli* proteins display negative log2 ratios, with the higher dilution (1:50) distinctly separated from the lower dilutions. Hence, integrating this "requantify" algorithm expands the coverage of DIA. Even though the additional data are of lower quality, they constitute valuable additional information that would otherwise be discarded.

While we mainly relied on the free software DIA-NN, we also tested DIA-SiS using Spectronaut. Here too, DIA-SiS increased protein identification, especially for low abundant proteins (supplemental Fig. S8). However, while the quantitative performance of DIA-SiS was better for the human proteins, LFQ outperforms DIA-SiS for the quantification of *E. coli* proteins. Importantly, the separation of the different *E. coli* protein abundance ratios is considerably better in our DIA-NN-based workflow than in the corresponding Spectronaut analysis of the same data (compare Fig. 2, *B* and *C* to supplemental Fig. S8, *C* and *D*). Hence, DIA-NN appears to outperform Spectronaut for DIA-SiS-based relative quantification. Investigating the reasons for these differences between DIA-NN and Spectronaut is beyond the scope of this manuscript. Generally, those results highlight the value of the ground truth dataset we provide for further use, including for the assessment of DIA software packages.

Missing values pose a significant challenge in quantitative proteomics (40, 41, 42). How to interpret missing values depends on whether a protein escapes detection due to random or technical factors (“missing at random”) or due to its inherently low abundance in a sample (“missing not at random”). In other words, the key question is whether identifying a protein in some samples but not in others should be interpreted as evidence for its lower abundance in the latter. With DDA data, missing LFQ values are typically interpreted as “missing not at random” and therefore imputed. A widely used approach is to use simulated values forming a distribution around the detection limit of actually measured intensities (8). Although DIA offers improved data completeness compared to DDA, the issue of missing values persists.

To evaluate the performance of DIA-SiS and LFQ in handling missing values, we conducted an additional benchmark study where one sample contained *E. coli* while the other did not. DIA-SiS (with "requantify" enabled) consistently identified more human and *E. coli* proteins than LFQ (Fig. 3*D*). As expected, the differences were especially pronounced for *E. coli* proteins, with DIA-SiS identifying over five times more across sample ratios (2507 *versus* 441) across all four replicates. Importantly, missing values also impacted human proteins (18% missing values with LFQ), despite their identical abundance in both samples. This issue is mitigated using SILAC (13.2% missing values) and further improved with SILAC + "requantify" (8.6% missing values; Fig. 3*E*). Interestingly, comparing the human proteins missing in LFQ but covered by DIA-SiS + "requantify" to the overall protein intensity distribution reveals that not all of the proteins gained by DIA-SiS + "requantify" are actually of low abundance (Fig. 3*F*). We note that imputing these missing values using a distribution around the detection limit of measured intensities would lead to the erroneous conclusion that they are downregulated in one sample. With DIA-SiS and "requantify", we significantly reduce the number of missing values and thereby avoid the need for imputation for most proteins. Importantly, across sample ratios for the vast majority of *E. coli* proteins (completely missing in one sample, Fig. 3*G*) as well as human proteins (not changing, independent of the benchmark, Fig. 3, *B*, *C*, and *G*) showed the correct trend. Therefore, DIA-SiS substantially alleviates the missing value problem.

### DIA-SiS Increases Identifications for Single Cell-like Amounts

Since our data indicate that DIA-SiS is particularly beneficial in low-input settings, we wanted to test its utility for the emerging field of single-cell proteomics (43). Therefore, we tested DIA-SiS using single cell-like amounts on the ultrahigh sensitivity mass spectrometer timsTOF SCP (44). For this purpose, we used the same benchmark samples as before, diluting them so that the maximum sample injection amount (sample S3) was 300 pg per species. As previously, the other samples contained less *E. coli* (supplemental Fig. S9). This time, we added different amounts of spike-in (2×, 5×, and 20× relative to the maximum light sample amount) in order to assess the optimal amount of spike-in. To enhance the coverage for these challenging low-input samples, “requantify” was used by default.

As expected, we observed an increased protein coverage with increasing spike-in amounts (Fig. 4*A*). The 2× spike-in already doubles *E. coli* protein identifications at the highest dilution (S1). The 5× and 20× spike-in further increase protein identifications. For instance, the 20× spike-in nearly doubles the human protein identifications compared to LFQ, which also yields the highest number of across-sample ratios (supplemental Fig. S10). On the other hand, as also expected, increasing the excess of heavy spike-in results in increased ratio compression (reduced accuracy) and decreased precision, especially at the 20× excess level (Fig. 4*B* and supplemental Fig. S10*B*). This effect is less pronounced with lower spike-in amounts, where distinguishing different across-sample ratios is still possible. Based on these observations, we conclude that a 5× spike-in is a suitable compromise, enhancing coverage while maintaining good quantitative performance. Consequently, we proceeded with the analysis of the 5× spike-in samples to identify differentially abundant proteins (Fig. 4*C*). As anticipated, the data for single cell-like amounts is generally noisier compared to higher input amounts (see Fig. 2*D* for comparison). Nevertheless, DIA-SiS results in a higher number of precursors per protein (supplemental Fig. S11) and identifies more differentially abundant proteins than LFQ in most comparisons (Fig. 4*C*). Importantly, the number of false positive hits (*i.e.*, human proteins passing *p*-value and log2FC cutoffs) does not increase and remains similar between LFQ and DIA-SiS. Additionally, many *E. coli* proteins that do not pass the *p*-value cutoff are still correctly categorized based on their log2FC in the DIA-SiS data, suggesting that this may be the preferred criterion for differential abundance analysis. We conclude that for ng-scale experiments, DIA-SiS can offer a substantial increase in protein identifications with modest ratio compression. Therefore, it is an attractive option for single-cell proteomics applications.

### Applying DIA-SiS to Low Input Tumor Samples

Encouraged by our ground-truth data, we aimed to evaluate DIA-SiS in a real-world scenario. Our rationale was to capitalize on the enhanced coverage achieved by DIA-SiS to improve identification of proteins in low-input samples. For this purpose, we investigated FFPE samples from HNSCCs.

Following the Super-SILAC concept (31), we used SILAC to label six HNSCC cell lines (supplemental Fig. S1*B*) and combined their lysates to create a mixed heavy spike-in reference. Unlike the benchmark samples involving two species discussed previously, no ground truth data are available for the FFPE samples. Consequently, we cannot use the FFPE samples (or any other biological samples) to benchmark the performance of DIA-SiS against ground truth data. Instead, we relied on the results of an LFQ analysis performed with standard input amounts and evaluated their recovery in low-input analyses both with and without the heavy spike-in. Specifically, we analyzed injections of 300 ng (standard input), 50 ng (low input), and 50 ng + spike-in (low input + SiS + “requantify”) for two HNSCC FFPE samples. All samples were measured in triplicates and examined using LFQ (300 ng and 50 ng) or our DIA-SiS pipeline with requantification enabled (50 ng + "requantify"). This approach allowed us to assess whether DIA-SiS can improve the coverage of lower input samples.

As expected, for both FFPEs, we observed the least number of protein groups in the 50 ng input samples (supplemental Fig. S12*A*). Briefly, 300 ng input samples increased the coverage significantly. However, most identifications were made with the 50 ng + spike-in + “requantify” approach, surpassing the 300 ng LFQ samples. The majority (96%) of protein groups identified with 50 ng input material have been identified in replicates of the other samples (supplemental Fig. S12*B*).

Reducing the input from 300 ng to 50 ng also significantly decreased the number of across sample ratios between two FFPE samples that could be quantified (Fig. 5*A*). Adding the heavy spike-in to the low input sample largely recovered the missing protein ratios, increasing the coverage to approximately 4500. About 90% of these ratios overlap with the ratios obtained with standard input samples. In order to verify the relevance of the additional IDs, we performed a differential abundance analysis on the 300 ng samples, labeling all proteins with a *p*-value <0.01 as upregulated or downregulated, depending on their FC. Subsequently, 70% of these significantly differentially abundant proteins are also identified in the 50 ng samples, while adding the spike-in increased the coverage to 86% (Fig. 5*B*). Reassuringly, the correlation of log2FCs in the standard input LFQ sample and the low input DIA-SiS sample was high for these differentially abundant proteins (R = 0.71, Fig. 5*C*). Additionally, we observed an increased precursor coverage per protein using 50 ng + SiS + “requantify” that is similar to the increase observed using 300 ng (supplemental Fig. S12*C*). Disabling “requantify” does lead to less coverage (supplemental Figs. S12 and S13) and fewer differentially abundant proteins but similar quantitative performance as with “requantify”. However, even without “requantify”, the coverage is higher than in the 50 ng sample without spike-in.

We conclude that DIA-SiS enhances the proteomic coverage of low-input human tissue samples, especially using “requantify”, thereby presenting a viable option for translational investigations.

## Conclusions

Although stable isotope labeling typically offers superior quantitative performance, most DIA studies to date have used LFQ due to its simplicity and broad applicability. In contrast, chemical stable isotope labeling techniques such as mass differential tags for relative and absolute quantification and dimethyl labeling require additional steps, and metabolic labeling *via* SILAC is limited to cell culture studies. Here, we demonstrate that integrating SiS with DIA combines the superior quantification of stable isotope labeling with the simplicity of label-free sample preparation, enabling straightforward and precise proteome profiling with improved coverage, especially for low-abundance proteins.

Interestingly, for the identification of differentially abundant proteins, we observe that the main advantage of DIA-SiS over LFQ is a better quantification of log2FCs but not necessarily *t* test *p*-values. More specifically, we observe that most proteins passing a log2FC of 1 are correct hits, even when their *t* test *p*-values are not significant. This effect is particularly pronounced in the single cell-like amount (Fig. 4*C*) and but also visible in bulk samples (Fig. 2*D*). The precision recall analysis also reveals the superiority of the log2 FC as a selection criterion (Fig. 2*E*). This suggests that to identify differentially abundant proteins using DIA-SiS, it may be best to mainly rely on the observed log2FC and to relax *p*-value cutoffs. Conversely, we find that the LFQ analysis can yield *p*-values that are too optimistic, which results in misclassification of numerous proteins as differentially abundant although they are in fact not changing. One limitation of DIA-SiS is that adding the heavy reference reduces the amount of light sample that can be injected into the mass spectrometer. This makes DIA-SiS especially attractive in low input scenarios, where the limited capacity is not of concern and the gains are particularly pronounced. For example, DIA-SiS is an attractive method for single cell proteomics (43). In such low-input conditions, the simple sample preparation process becomes an especially advantageous feature (11, 12). Indeed, our benchmark experiments with single cell-like amounts demonstrate that DIA-SiS significantly enhances proteome coverage while maintaining good quantitative performance. However, although coverage improves with increasing spike-in amounts, excessive spike-in can lead to reduced quantitative accuracy and precision (Fig. 4). Considering this tradeoff, we recommend a 5× excess of spike-in as a good compromise in a low-input setting.

While the ease of SILAC makes it the preferred method for cell culture experiments in DIA, obtaining a suitable heavy reference for human tissue samples can be more challenging. Looking ahead, the broader availability of synthetic proteomes may ease this process (45). Moreover, a further advantage of SILAC is its capability to study proteome turnover. This is an area where the reliable quantification of low-abundant proteins can be instrumental. While we did not explore this topic here, it can be expected to benefit substantially from increased coverage and quantitative performance that DIA-SiS can offer.

## Data and Code Availability

The mass spectrometry proteomics data and the R scripts for data analysis and generation of the plots for this manuscript have been deposited to the ProteomeXchange Consortium *via* the PRIDE (46) partner repository with the dataset identifiers PXD052080 and PXD054343. Additionally, we provide an R and Python package for the DIA-SiS pipeline in our lab github repository (https://github.com/SelbachLab).

## Supplemental data

This article contains supplemental data.

## Conflict of interest

P. M. and M. S. are Editorial Board Members/Editor-in-Chief/Associate Editors/Guest Editors for *Molecular and Cellular Proteomics* and were not involved in the editorial review or the decision to publish this article. The other authors declare no competing interests.
