# Supplementary material for: Combining Data Independent Acquisition With Spike-In SILAC (DIA-SiS) Improves Proteome Coverage and Quantification
Source: Mol Cell Proteomics. 2024 Sep 11;23(10):100839. doi: 10.1016/j.mcpro.2024.100839 (PMC11795695; doi:10.1016/j.mcpro.2024.100839)
Supplement: Supplemental data [file mmc1.pdf]

# Combining Data Independent Acquisition with Spike-in SILAC (DIA-SiS) Improves Proteome Coverage and Quantification

Anna Sophie Welter[1,2]*, Maximilian Gerwien[1,2]*, Robert Kerridge[1,2], Keziban Merve Alp[3], Philipp Mertins[3,4], Matthias Selbach[1,5]

[1] Max Delbrück Center for Molecular Medicine, Proteome Dynamics, Berlin, Germany
[2] Faculty of Life Sciences, Humboldt-Universität zu Berlin, Berlin, Germany
[3] Max Delbrück Center for Molecular Medicine, Proteomics, Berlin, Germany
[4] Berlin Institute of Health, Core Unit Proteomics, Berlin, Germany
[5] Charité - Universitätsmedizin Berlin, Berlin, Germany

* these authors contributed equally to the publication

# Supplemental Information

# Contents


- Figure S1: Labeling efficiency of the SILAC spike-ins.
- Figure S2: Precursor coverage comparison between LFQ (green) and DIA-SiS (purple).
- Figure S3: Maximum loading amounts.
- Figure S4: Quantitative performance of LFQ and DIA-SiS.
- Figure S5: Comparison of directLFQ (external package), LFQ (based on DIA-NN output) and DIA-SiS
- Figure S6: Quantification performance of DIA-SiS-exclusive protein groups
- Figure S7: Across sample protein quantification of proteins with the exact same number of precursors passing filters
- Figure S8: Using Spectronaut to analyze the main benchmark data
- Figure S9: Single cell-like amount benchmark design
- Figure S10: Application of DIA-SiS to single cell-like amounts
- Figure S11: Precursor coverage comparison between LFQ (green) and 5x DIA-SiS + "requantify" (purple) of the single cell-like amounts benchmark data
- Figure S12: IDs, overlapping protein groups and sequence coverage for 300 ng, 50 ng and 50 ng + SiS and 50 ng + SiS + “requantify” FFPE samples
- Figure S13: Application of DIA-SiS (without “requantify”) to formalin-fixed paraffin-embedded (FFPE) head and neck squamous cell carcinoma (HNSCC) samples
- Pseudocode SILAC Ratio Calculation.

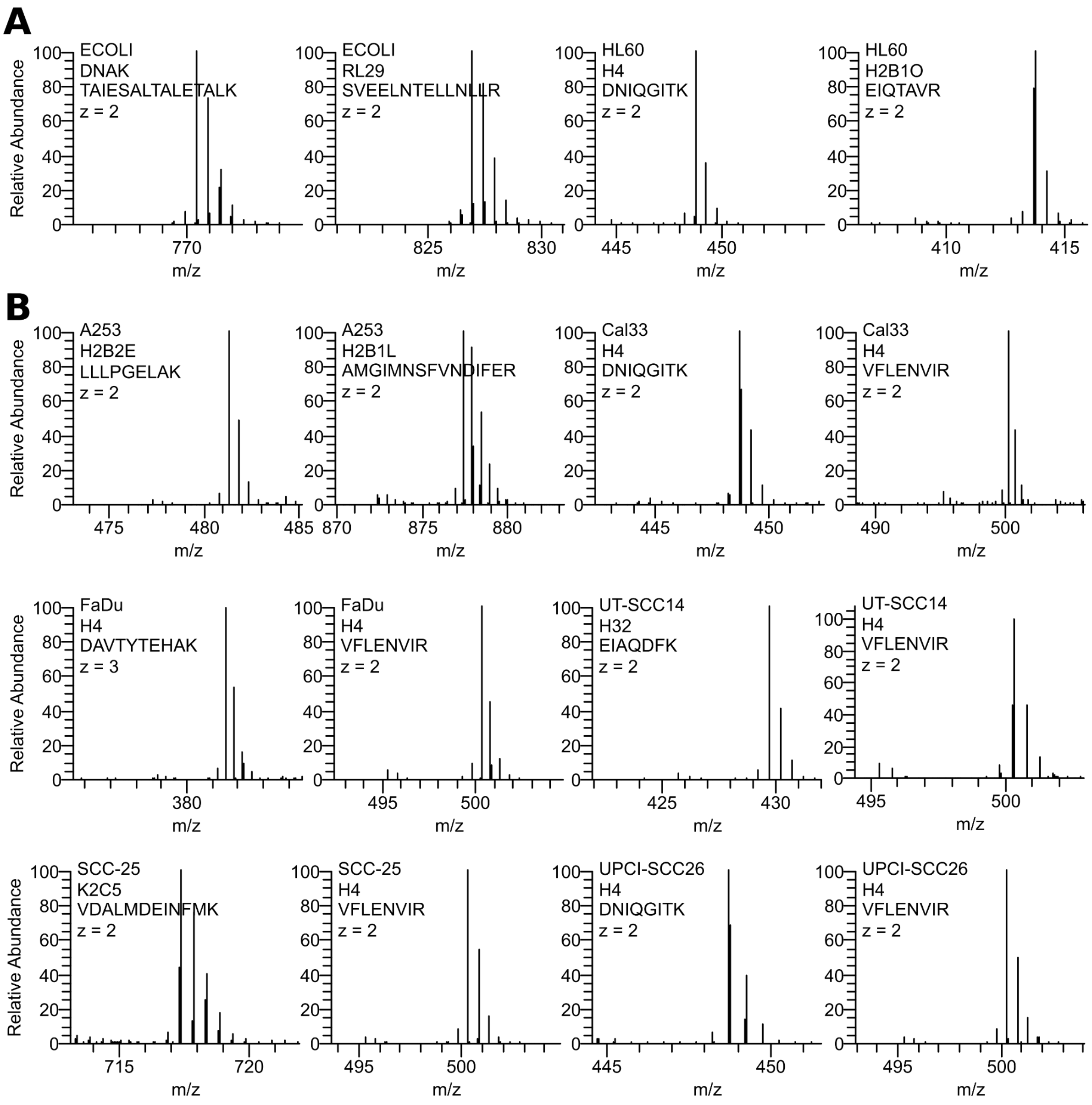


**Figure S1: Labeling efficiency of the SILAC spike-ins.**

Plots show the heavy (100 % relative abundance) and the corresponding light isotopic clusters for one high intensity arginine and lysine containing precursor of all heavy labeled cells used in **A)** the benchmarking experiments and **B)** the Super-DIA-SiS approach.

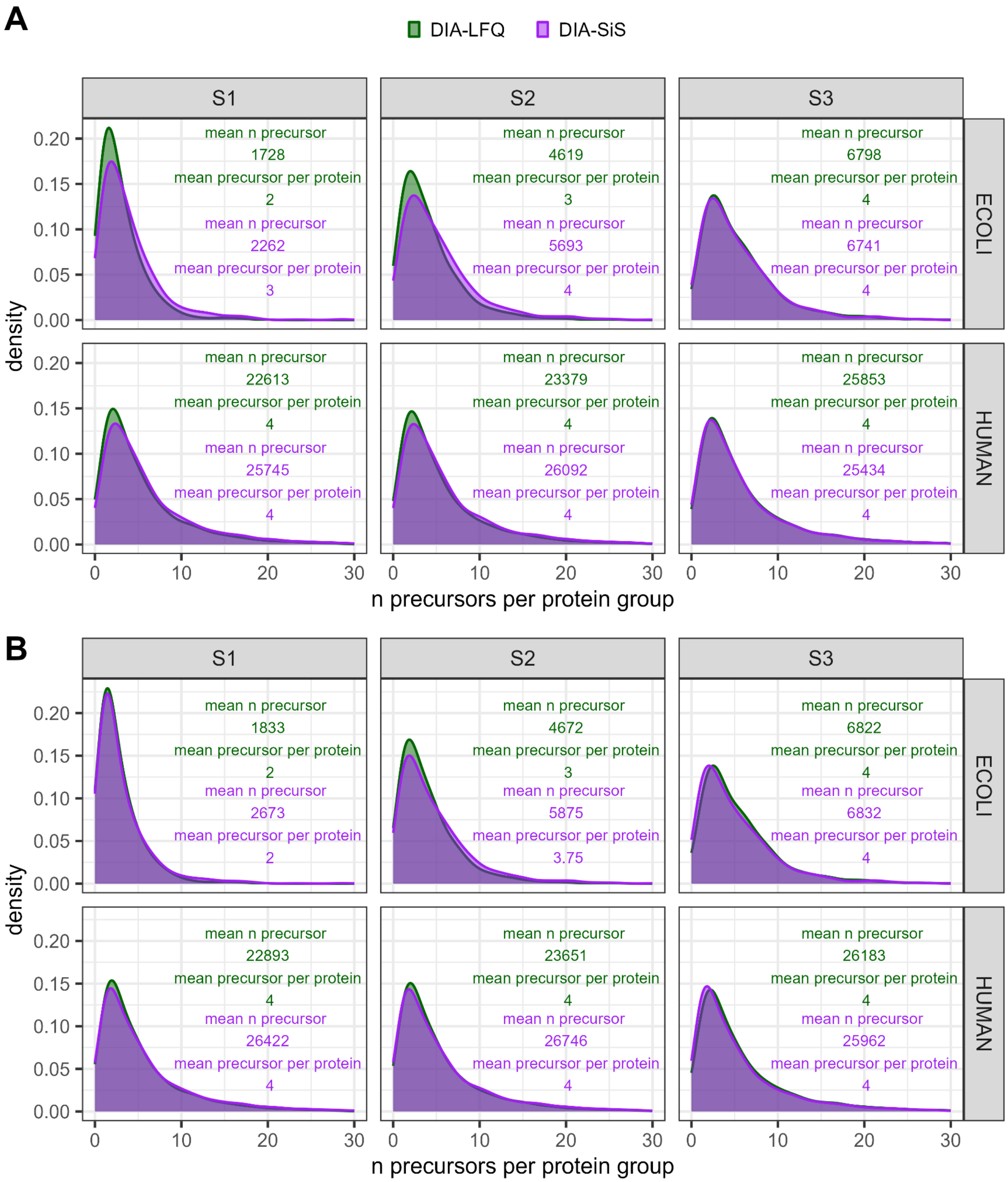
A
DIA-LFQ
DIA-SiS
S1
S2
S3
ECOLI
HUMAN
density
0.00
0.05
0.10
0.15
0.20
0
10
20
30
n precursors per protein group
mean n precursor
1728
mean precursor per protein
2
mean n precursor
2262
mean precursor per protein
3
mean n precursor
4619
mean precursor per protein
3
mean n precursor
5693
mean precursor per protein
4
mean n precursor
6798
mean precursor per protein
4
mean n precursor
6741
mean precursor per protein
4
mean n precursor
22613
mean precursor per protein
4
mean n precursor
25745
mean precursor per protein
4
mean n precursor
23379
mean precursor per protein
4
mean n precursor
26092
mean precursor per protein
4
mean n precursor
25853
mean precursor per protein
4
mean n precursor
25434
mean precursor per protein
4
B
S1
S2
S3
ECOLI
HUMAN
density
n precursors per protein group
mean n precursor
1833
mean precursor per protein
2
mean n precursor
2673
mean precursor per protein
2
mean n precursor
4672
mean precursor per protein
3
mean n precursor
5875
mean precursor per protein
3.75
mean n precursor
6822
mean precursor per protein
4
mean n precursor
6832
mean precursor per protein
4
mean n precursor
22893
mean precursor per protein
4
mean n precursor
26422
mean precursor per protein
4
mean n precursor
23651
mean precursor per protein
4
mean n precursor
26746
mean precursor per protein
4
mean n precursor
26183
mean precursor per protein
4
mean n precursor
25962
mean precursor per protein
4

**Figure S2: Precursor coverage comparison between LFQ (green) and DIA-SiS (purple).**

**A)** Intersection dataset. Intersection is based on overlapping protein groups between a LFQ and the corresponding DIA-SiS replicate. **B)** Complete dataset (no "requantify").
X axis indicates the number of precursors that identify a protein group. Numbers indicate the mean number of precursors identified over all 4 replicates per sample and the mean median number of precursors over all 4 replicates that identify that protein group. For both, LFQ and DIA-SiS only precursors that passed the q-value filtering were considered.

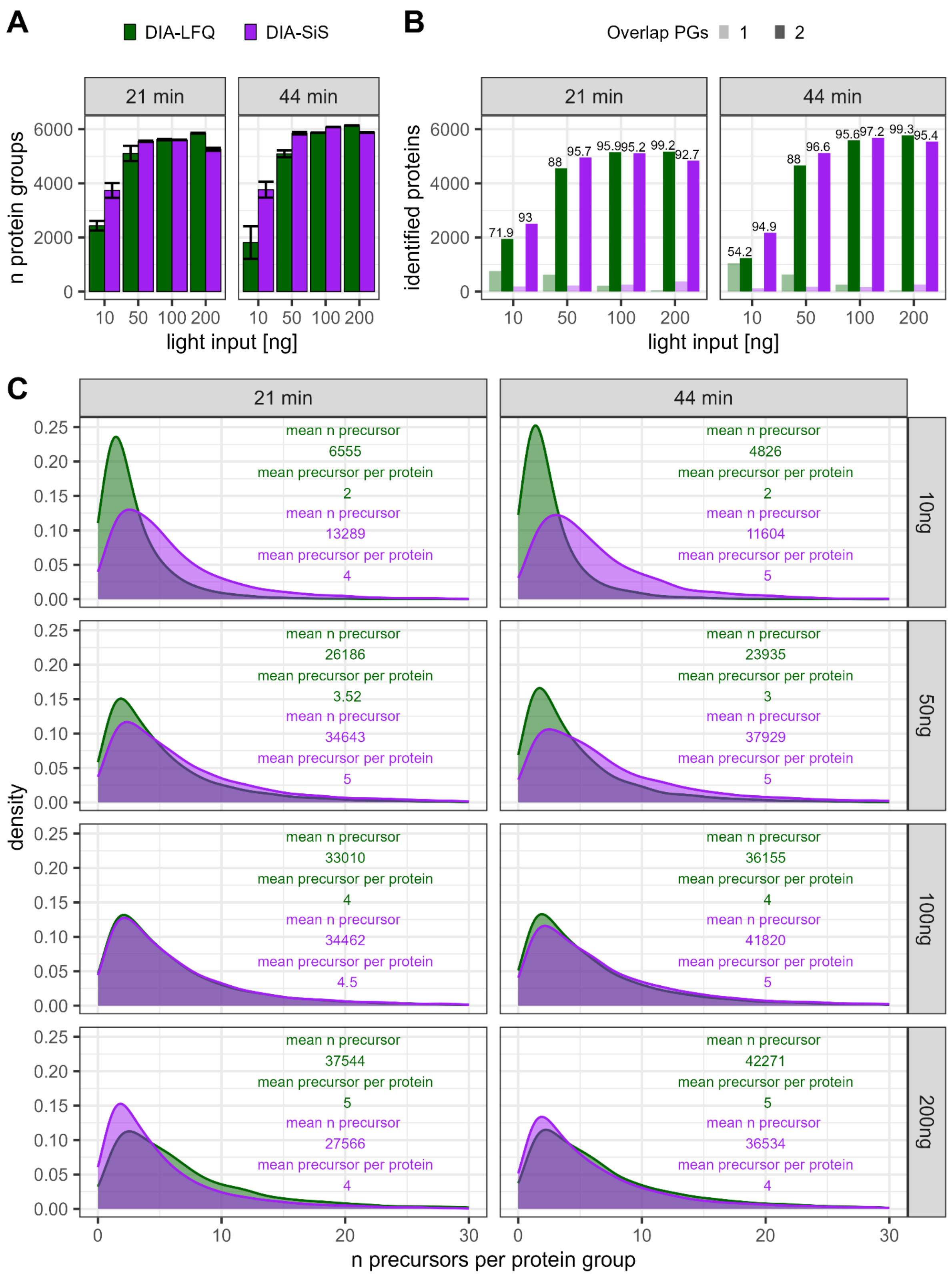
A
DIA-LFQ
DIA-SiS
21 min
44 min
n protein groups
0
2000
4000
6000
10 50 100 200
light input [ng]
B
Overlap PGs 1 2
identified proteins
71.9
93
88
95.7
95.9
95.2
99.2
92.7
54.2
94.9
96.6
95.6
97.2
99.3
95.4
C
density
n precursors per protein group
10ng
50ng
100ng
200ng
mean n precursor
6555
mean precursor per protein
2
mean n precursor
13289
mean precursor per protein
4
mean n precursor
4826
mean precursor per protein
2
mean n precursor
11604
mean precursor per protein
5
mean n precursor
26186
mean precursor per protein
3.52
mean n precursor
34643
mean precursor per protein
5
mean n precursor
23935
mean precursor per protein
3
mean n precursor
37929
mean precursor per protein
5
mean n precursor
33010
mean precursor per protein
4
mean n precursor
34462
mean precursor per protein
4.5
mean n precursor
36155
mean precursor per protein
4
mean n precursor
41820
mean precursor per protein
5
mean n precursor
37544
mean precursor per protein
5
mean n precursor
27566
mean precursor per protein
4
mean n precursor
42271
mean precursor per protein
5
mean n precursor
36534
mean precursor per protein
4

**Figure S3: Maximum loading amounts.**

Additional human-only benchmark measured on the timsTOF Pro 2 with either a 21 min gradient (left) or a 44 min gradient (right; as the main benchmark) and different light input amounts (10, 50, 100, 200 ng) and always double the amount of spike-in. Samples are technical duplicates; LFQ data is green, DIA-SiS is purple. **A)** Mean number of identified protein groups using either LFQ or DIA-SiS (2x spike-in) over both technical replicates. **B)** Number of identified proteins in one or both replicates (increasing opacity) of the intersecting proteins. Numbers indicate the percentage of human protein groups detected in both replicates. **C)** Precursor coverage using the different input amounts and gradients of the overlapping protein group.

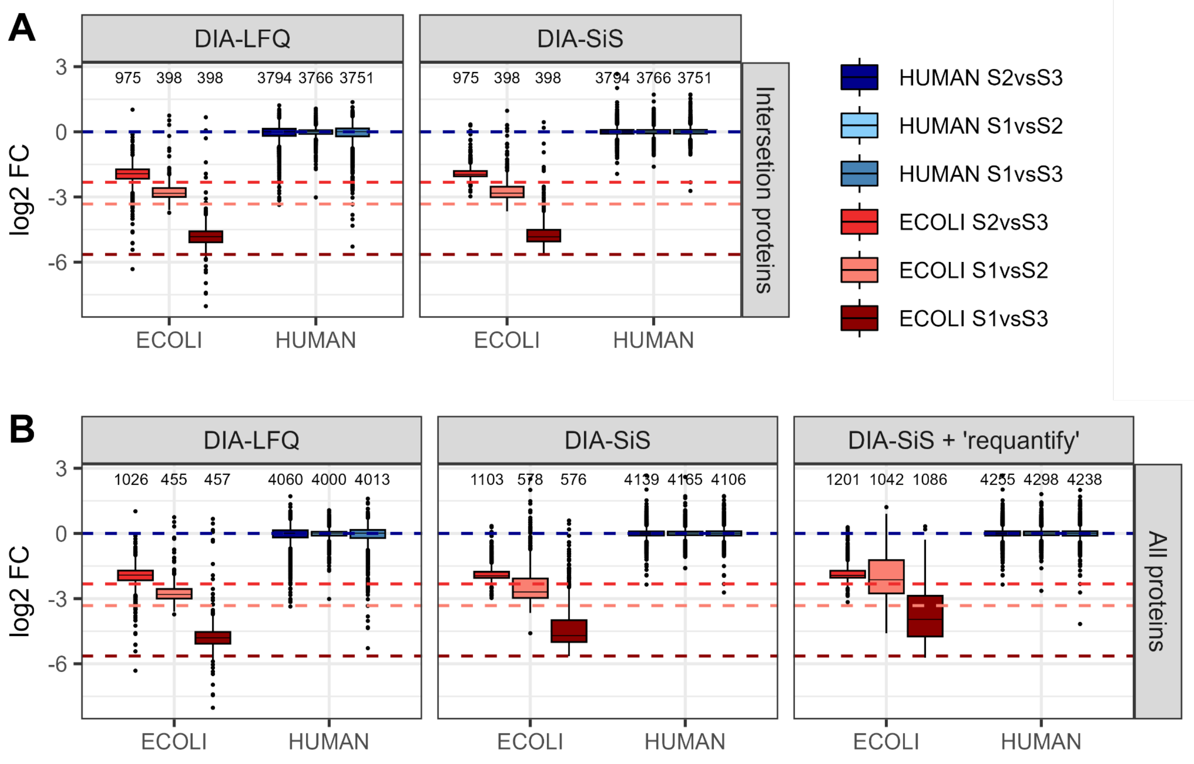


**Figure S4: Quantitative performance of LFQ and DIA-SiS.**

All proteins with no missing values across all replicates are shown. Numbers indicate the number of protein ratios displayed. **A**) log2FC boxplots of intersection proteins between LFQ and DIA-SiS. **B)** log2FC boxplots of all proteins detected in all 4 replicates per dilution with LFQ, DIA-SiS and DIA-SiS + "requantify".

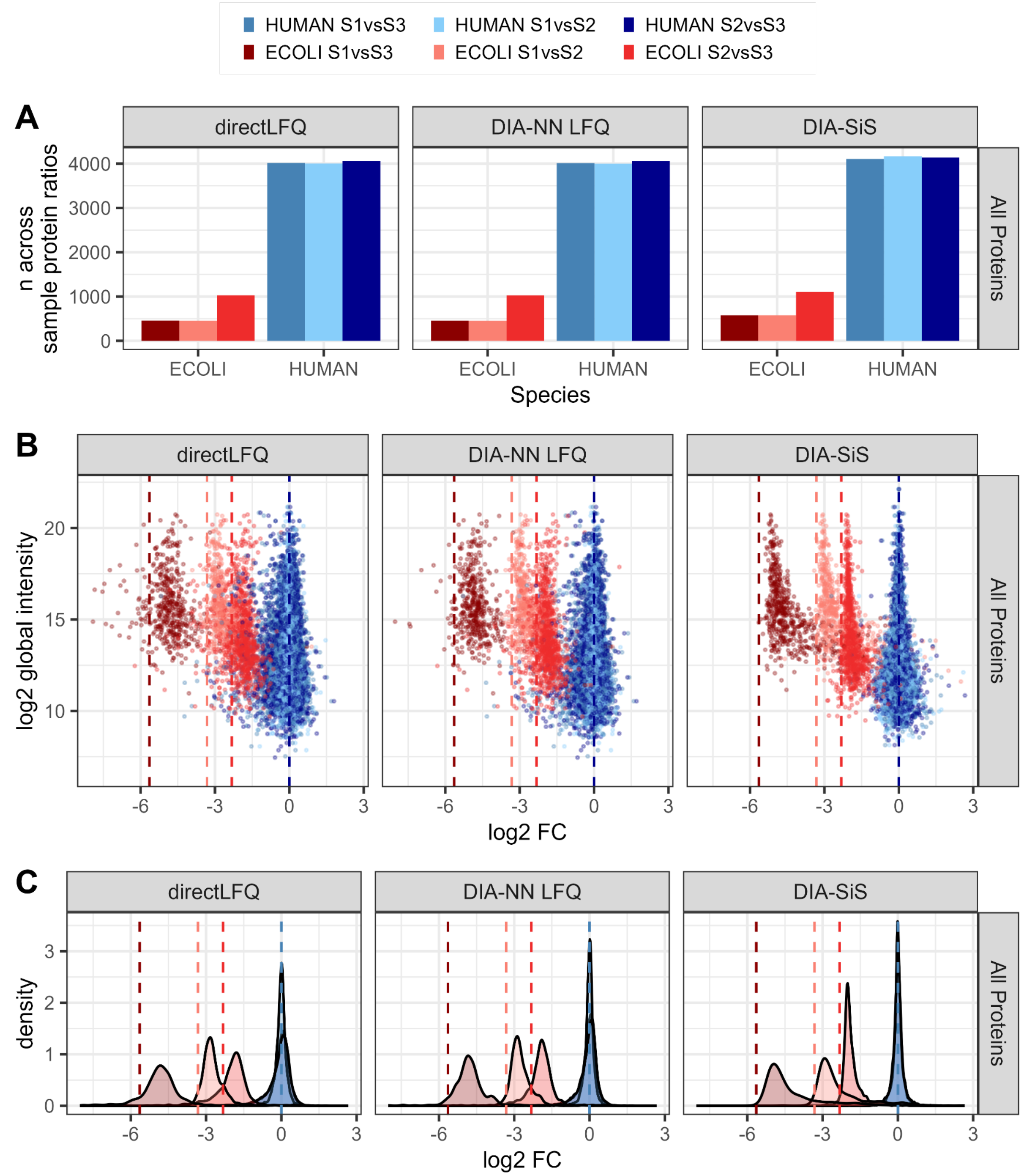

HUMAN S1vsS3
HUMAN S1vsS2
HUMAN S2vsS3
ECOLI S1vsS3
ECOLI S1vsS2
ECOLI S2vsS3
A
directLFQ
DIA-NN LFQ
DIA-SiS
All Proteins
n across sample protein ratios
0
1000
2000
3000
4000
ECOLI
HUMAN
Species
B
log2 global intensity
10
15
20
-6
-3
0
3
log2 FC
C
density
0
1
2
3
log2 FC

**Figure S5: Comparison of directLFQ (external package), LFQ (based on DIA-NN output) and DIA-SiS.**

All proteins with no missing values across all replicates are shown. **A**) The bars indicate the number of across-sample protein ratios quantified with both LFQ and DIA-SiS. **B)** directLFQ, LFQ from DIA-NN and DIA-SiS capture the expected across-sample ratios, with a clearer intensity-dependent precision for DIA-SiS. The global protein group abundance is plotted against the mean across-sample protein ratios. Dashed lines indicate expected ratios. **C)** Density plots corresponding to B).

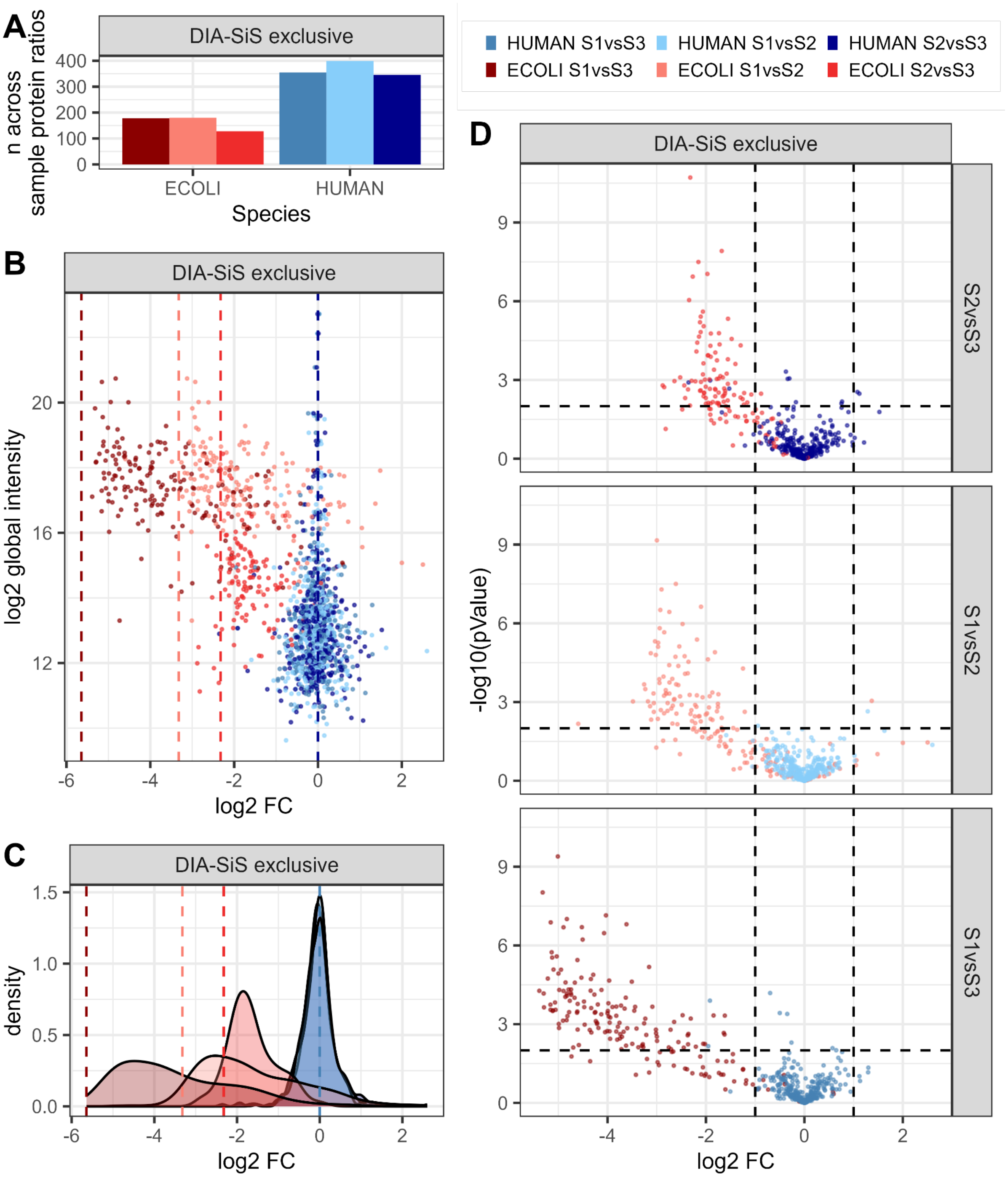

A
DIA-SiS exclusive
n across sample protein ratios
400
300
200
100
0
ECOLI
HUMAN
Species
HUMAN S1vsS3
HUMAN S1vsS2
HUMAN S2vsS3
ECOLI S1vsS3
ECOLI S1vsS2
ECOLI S2vsS3
B
DIA-SiS exclusive
log2 global intensity
20
16
12
-6
-4
-2
0
2
log2 FC
C
DIA-SiS exclusive
density
1.5
1.0
0.5
0.0
log2 FC
D
DIA-SiS exclusive
-log10(pValue)
9
6
3
0
S2vsS3
S1vsS2
S1vsS3
log2 FC

**Figure S6: Quantification performance of DIA-SiS-exclusive protein groups.**

Only proteins with DIA-SiS-exclusive ratios, no missing values across all replicates are shown. **A**) Set size, number of Human (blue) and *E. coli* (red) across-sample protein ratios; **B)** Mean across-sample protein ratios (y-axis) versus global protein group abundance (x-axis). Dashed lines indicate p value cutoffs of > 0.01 and a log2FC of > 1 / < -1, human proteins in blue, *E. coli* in red. **C)** Density of mean across-sample protein ratios (x-axis) corresponding to plots in B); **D)** -log10 p values (y-axis) versus mean across sample ratios (x-axis) for DIA-SiS and three different target ratios (S2vsS3 1:5, S1vsS2 1:10, S1vsS3 1:50).

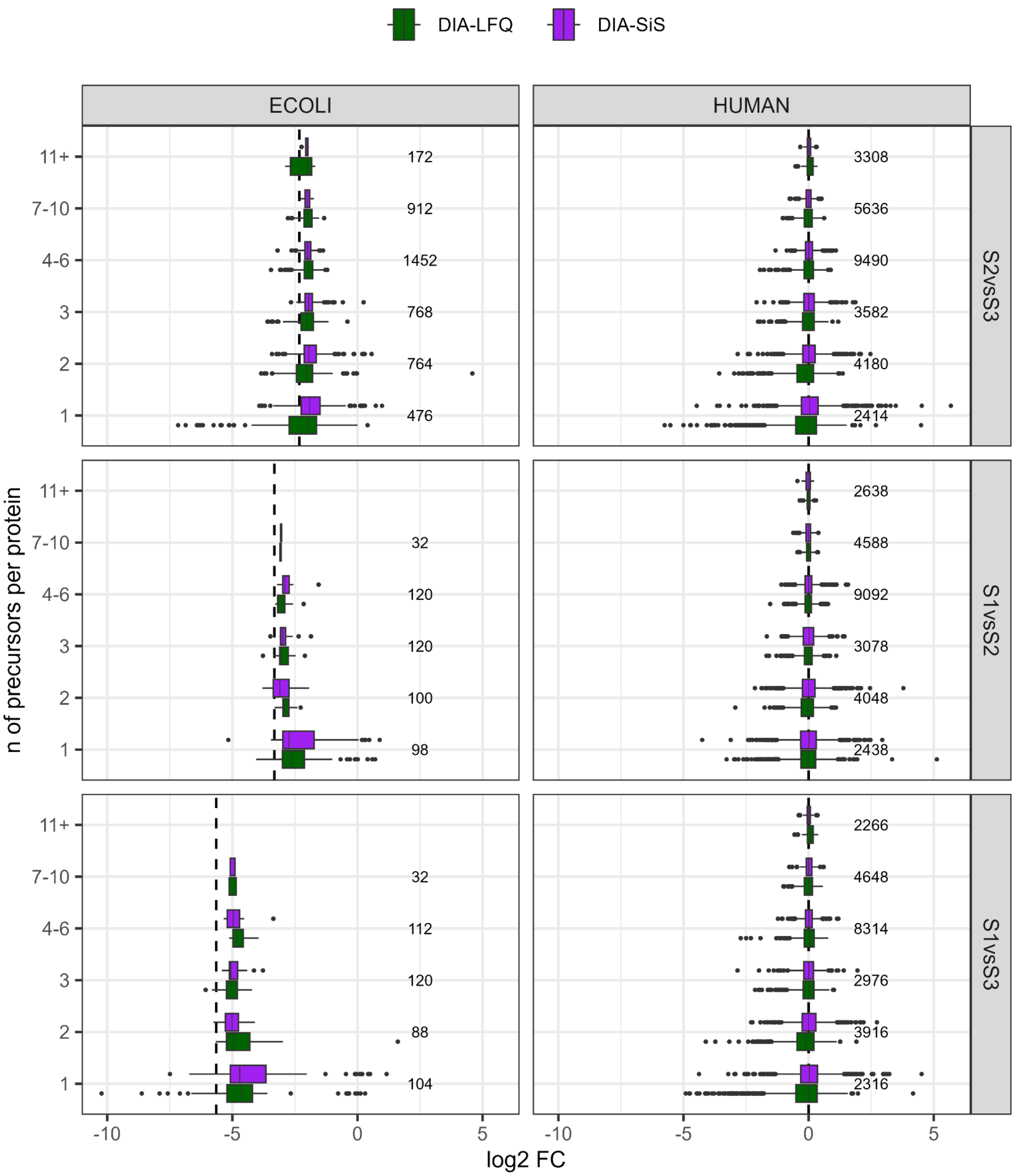

DIA-LFQ
DIA-SiS
ECOLI
HUMAN
S2vsS3
S1vsS2
S1vsS3
n of precursors per protein
log2 FC
11+
7-10
4-6
3
2
1
172
912
1452
768
764
476
3308
5636
9490
3582
4180
2414
32
120
120
100
98
2638
4588
9092
3078
4048
2438
32
112
120
88
104
2266
4648
8314
2976
3916
2316
-10
-5
0
5

**Figure S7: Across sample protein quantification of proteins with the exact same number of precursors passing filters.**

Plot shows the binned number of precursors per protein (y axis) and the protein log2FC (x-axis) across samples for *E. coli* (left) and human (right) proteins per replicate per dilution. Dataset is reduced to proteins with the same number of precursors passing filters in both dilutions with the same number of precursors in LFQ and DIA-SiS per replicate.

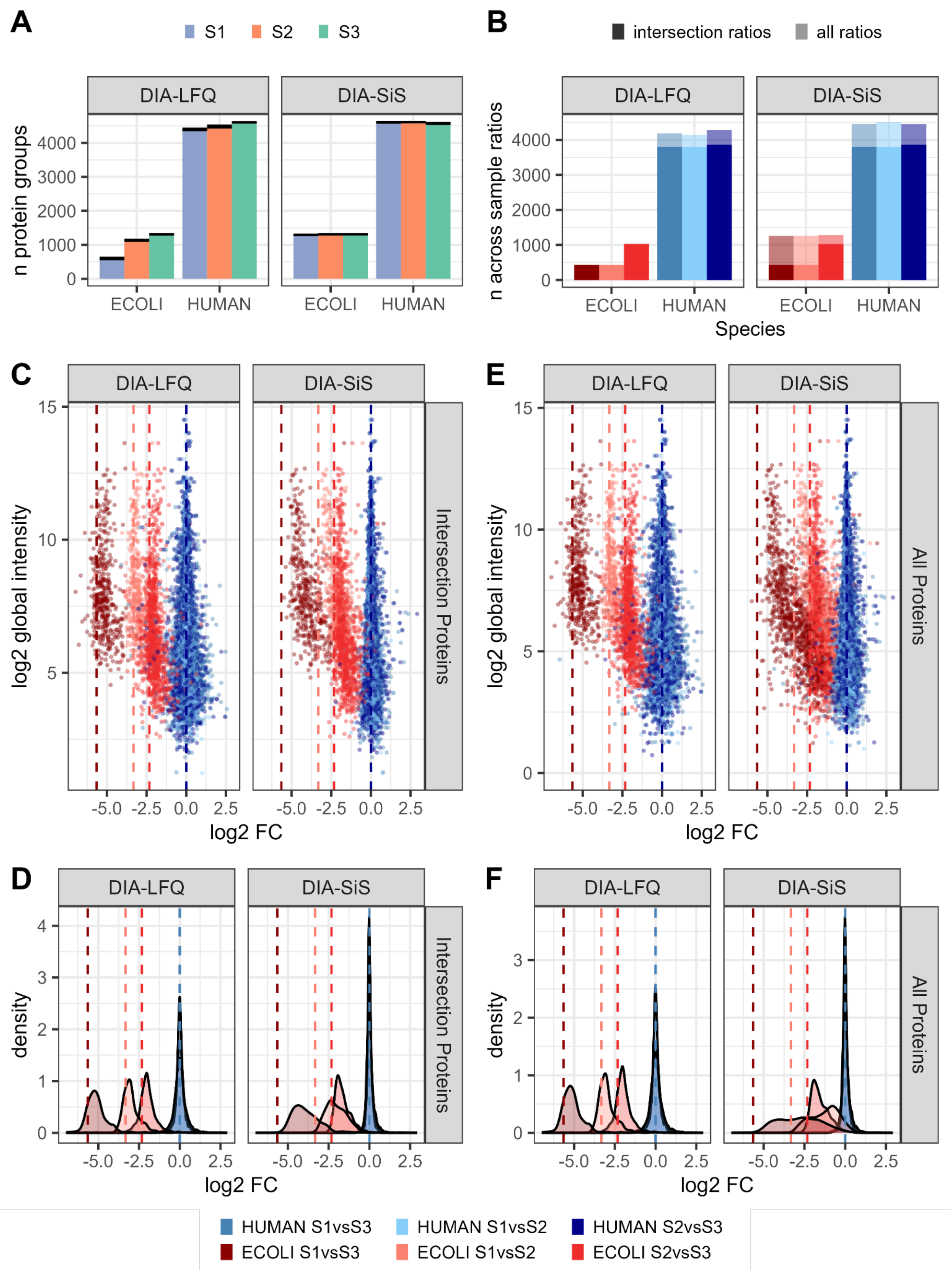

A
S1
S2
S3
DIA-LFQ
DIA-SiS
n protein groups
ECOLI
HUMAN
B
intersection ratios
all ratios
n across sample ratios
Species
C
log2 global intensity
log2 FC
Intersection Proteins
E
All Proteins
D
density
F
HUMAN S1vsS3
HUMAN S1vsS2
HUMAN S2vsS3
ECOLI S1vsS3
ECOLI S1vsS2
ECOLI S2vsS3

**Figure S8: Using Spectronaut to analyze the main benchmark data.**

The same benchmark as in the previous figures but analyzed with Spectronaut. **A)** Mean number of protein groups quantified per dilution (indicated by colors). Whiskers indicate standard deviation. **B**) Number of across sample ratios without missing values in any of the replicates. Dark colors indicate the number of ratios in the LFQ and DIA-SiS intersection set, light colors indicate the total number of ratios. **C-F)** The across sample protein ratios of intersecting (**C** and **D**) and all proteins (**E** and **F)**. Density plots correspond to the Christmas tree plots above.

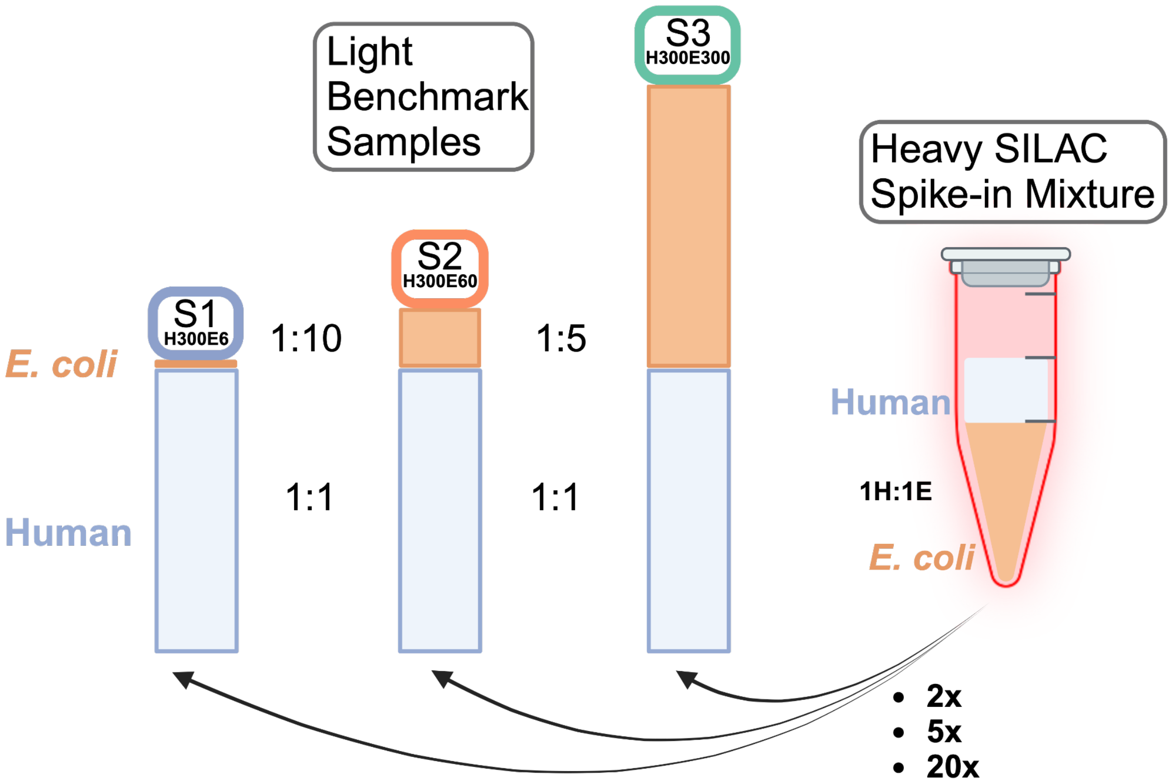


**Figure S9: Single cell-like amount benchmark design.**

The samples from Figure 1A were further diluted to render the sample set shown above. For S3, 300 pg human and 300 pg *E. coli* peptides were injected together with a spike-in mixture consisting of 1:1 human: *E. coli* heavy peptides. This spike-in mixture was added in a 2x, 5x or 20x excess relative to the lowest dilution, S3.

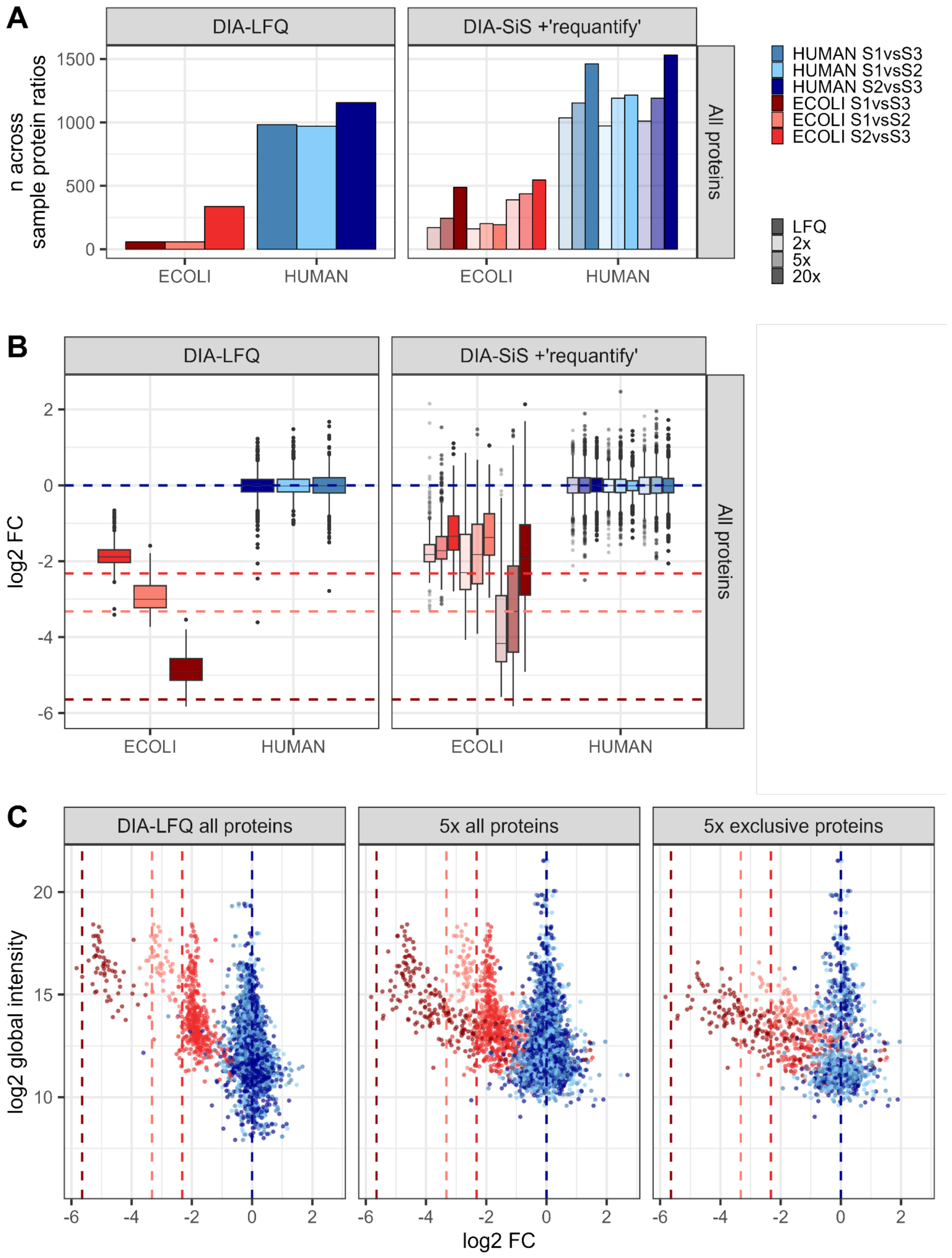
A
DIA-LFQ
DIA-SiS +'requantify'
All proteins
n across sample protein ratios
1500
1000
500
0
ECOLI
HUMAN
HUMAN S1vsS3
HUMAN S1vsS2
HUMAN S2vsS3
ECOLI S1vsS3
ECOLI S1vsS2
ECOLI S2vsS3
LFQ
2x
5x
20x
B
DIA-LFQ
DIA-SiS +'requantify'
All proteins
log2 FC
2
0
-2
-4
-6
ECOLI
HUMAN
C
DIA-LFQ all proteins
5x all proteins
5x exclusive proteins
log2 global intensity
20
15
10
-6
-4
-2
0
2
log2 FC

**Figure S10: Application of DIA-SiS to single cell-like amounts.**

The same benchmark as in the previous figures has been diluted in such a way that the lowest dilution contains 300 pg Human and 300 pg *E. coli* protein. All proteins with no missing values across all replicates are shown. Colors correspond to the different sample comparisons **A**) The bars indicate the number of across-sample protein ratios without spike-in (LFQ) and various amounts of spike-in (2x, 5x and 20x). For DIA-SiS data, the decreased transparency corresponds to the increasing spike-in amounts. **B)** Across-sample ratios of all proteins without spike-in (LFQ) and various amounts of spike-in (2x, 5x and 20x). For DIA-SiS data, the decreased transparency corresponds to the increasing spike-in amounts. **C)** Abundance vs. across-sample ratio of all LFQ proteins (left), all proteins in the 5x samples (middle) and the 5x exclusive proteins (right).

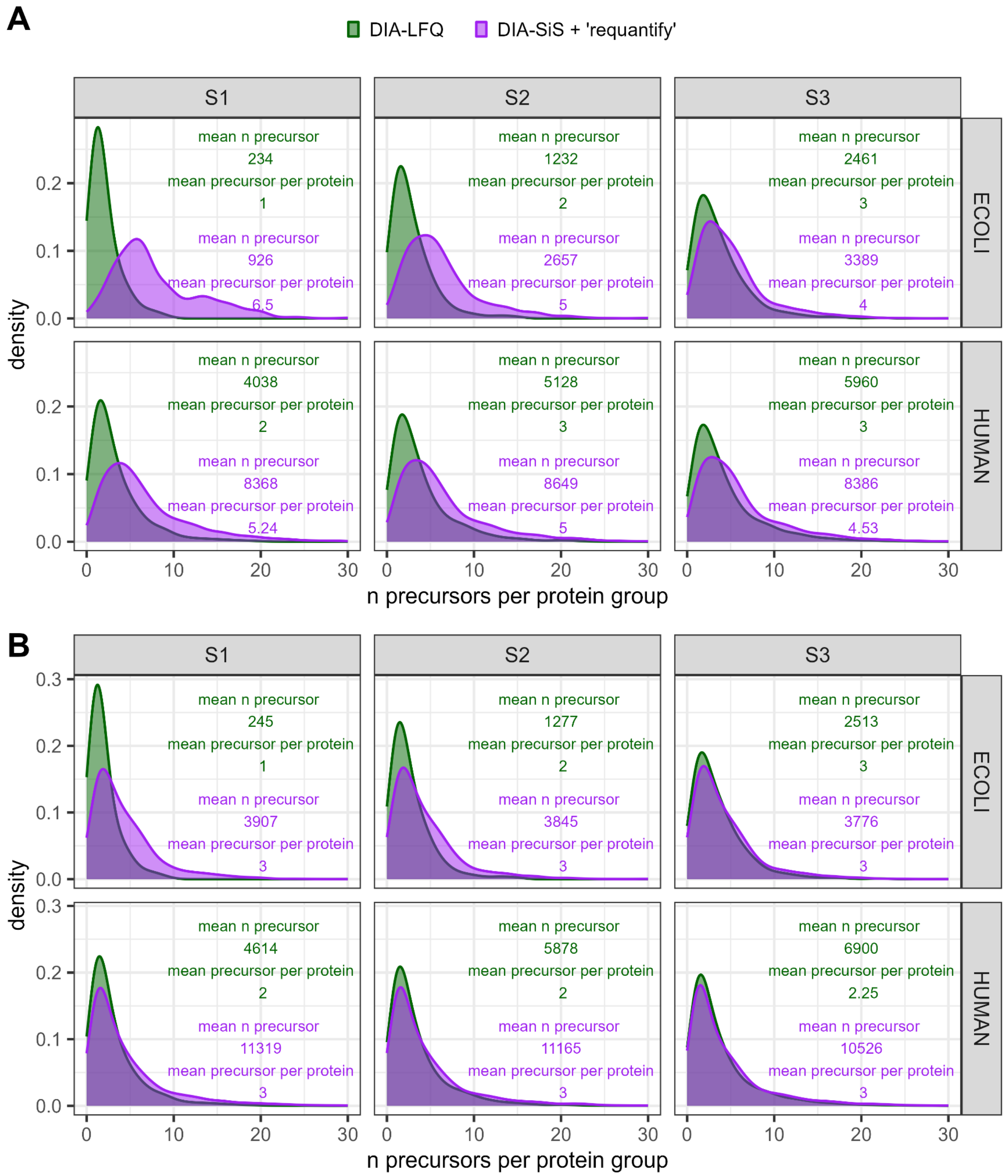

A
DIA-LFQ
DIA-SiS + 'requantify'
S1
S2
S3
ECOLI
HUMAN
density
mean n precursor
234
mean precursor per protein
1
mean n precursor
926
mean precursor per protein
6.5
mean n precursor
1232
mean precursor per protein
2
mean n precursor
2657
mean precursor per protein
5
mean n precursor
2461
mean precursor per protein
3
mean n precursor
3389
mean precursor per protein
4
mean n precursor
4038
mean precursor per protein
2
mean n precursor
8368
mean precursor per protein
5.24
mean n precursor
5128
mean precursor per protein
3
mean n precursor
8649
mean precursor per protein
5
mean n precursor
5960
mean precursor per protein
3
mean n precursor
8386
mean precursor per protein
4.53
n precursors per protein group
B
mean n precursor
245
mean precursor per protein
1
mean n precursor
3907
mean precursor per protein
3
mean n precursor
1277
mean precursor per protein
2
mean n precursor
3845
mean precursor per protein
3
mean n precursor
2513
mean precursor per protein
3
mean n precursor
3776
mean precursor per protein
3
mean n precursor
4614
mean precursor per protein
2
mean n precursor
11319
mean precursor per protein
3
mean n precursor
5878
mean precursor per protein
2
mean n precursor
11165
mean precursor per protein
3
mean n precursor
6900
mean precursor per protein
2.25
mean n precursor
10526
mean precursor per protein
3
n precursors per protein group

**Figure S11: Precursor coverage comparison between LFQ (green) and 5x DIA-SiS + "requantify" (purple) of the single cell-like amounts benchmark data.**

**A)** Intersection dataset. Intersection is based on overlapping protein groups between a LFQ and the corresponding DIA-SiS replicate. **B)** Complete dataset.
X axis indicates the number of precursors that identify a protein group. Numbers indicate the mean number of precursors identified over all 4 replicates per sample and the mean median number of precursors over all 4 replicates that identify that protein group. For both, LFQ and DIA-SiS only precursors that passed the q-value filtering were considered.

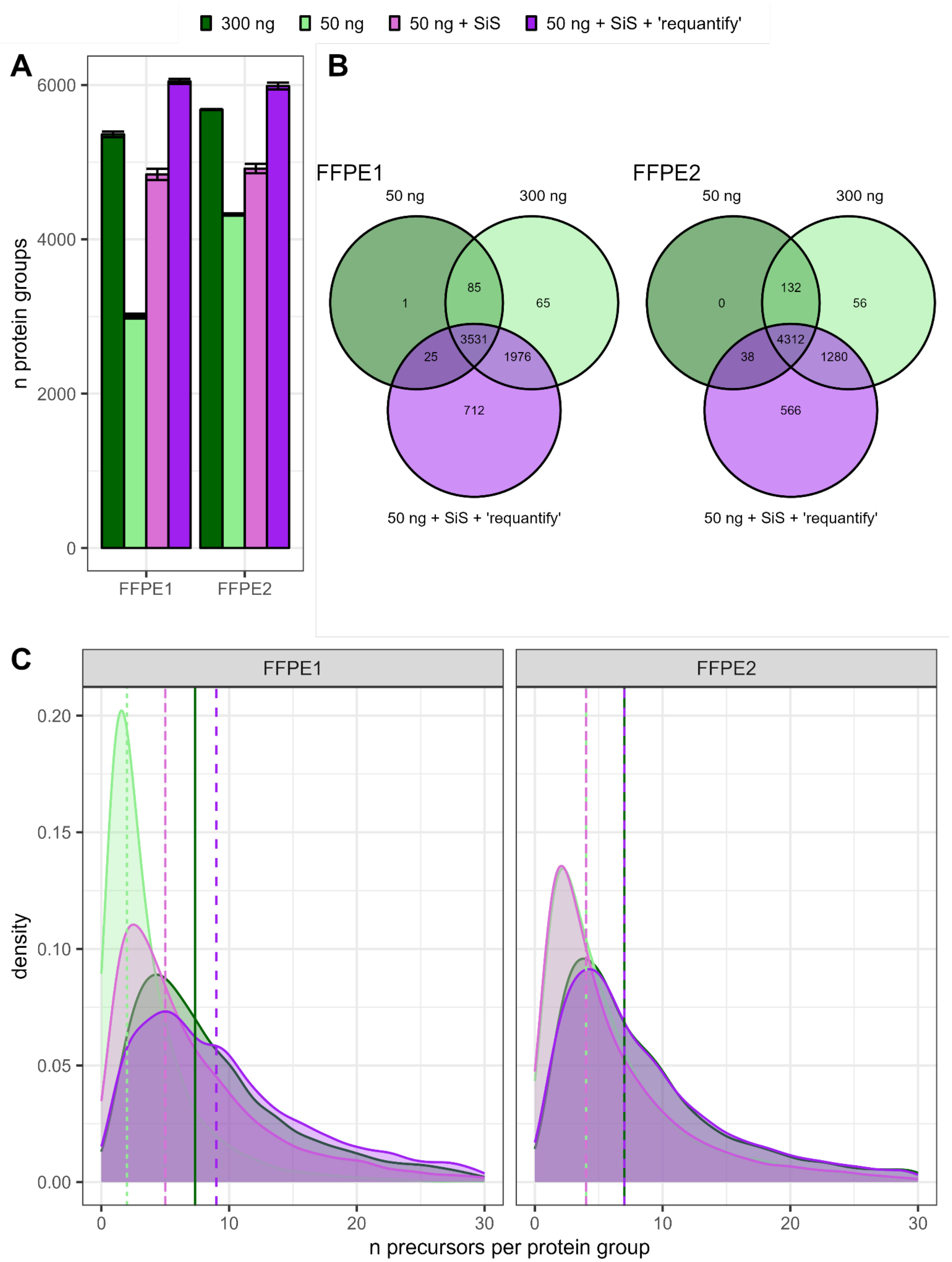

300 ng
50 ng
50 ng + SiS
50 ng + SiS + 'requantify'
A
n protein groups
6000
4000
2000
0
FFPE1
FFPE2
B
FFPE1
50 ng
300 ng
1
85
65
3531
25
1976
712
50 ng + SiS + 'requantify'
FFPE2
50 ng
300 ng
0
132
56
4312
38
1280
566
50 ng + SiS + 'requantify'
C
FFPE1
FFPE2
density
0.20
0.15
0.10
0.05
0.00
0
10
20
30
n precursors per protein group

**Figure S12: IDs, overlapping protein groups and sequence coverage for 300 ng, 50 ng and 50 ng + SiS and 50 ng + SiS + “requantify” FFPE samples.**

Supplemental data for the two analyzed FFPE samples and the different measurement approaches and sample amounts: 300 ng (dark green), 50 ng (light green), 50 ng + SiS (light purple), and 50 ng + SiS + “requantify” (purple). **A)** Mean number of quantified protein groups over 3 technical replicates. Whiskers indicate standard deviation. **B)** Overlap of protein groups between the three data sets per FFPE for all proteins detected at least in one technical replicate. DIA-SiS has a 100 % overlap with the "requantify" proteins and was excluded from the Venn. **C) P**recursor coverage using the different data sets of the intersecting proteins. Lines indicate the mean number of precursors per protein over all replicates.

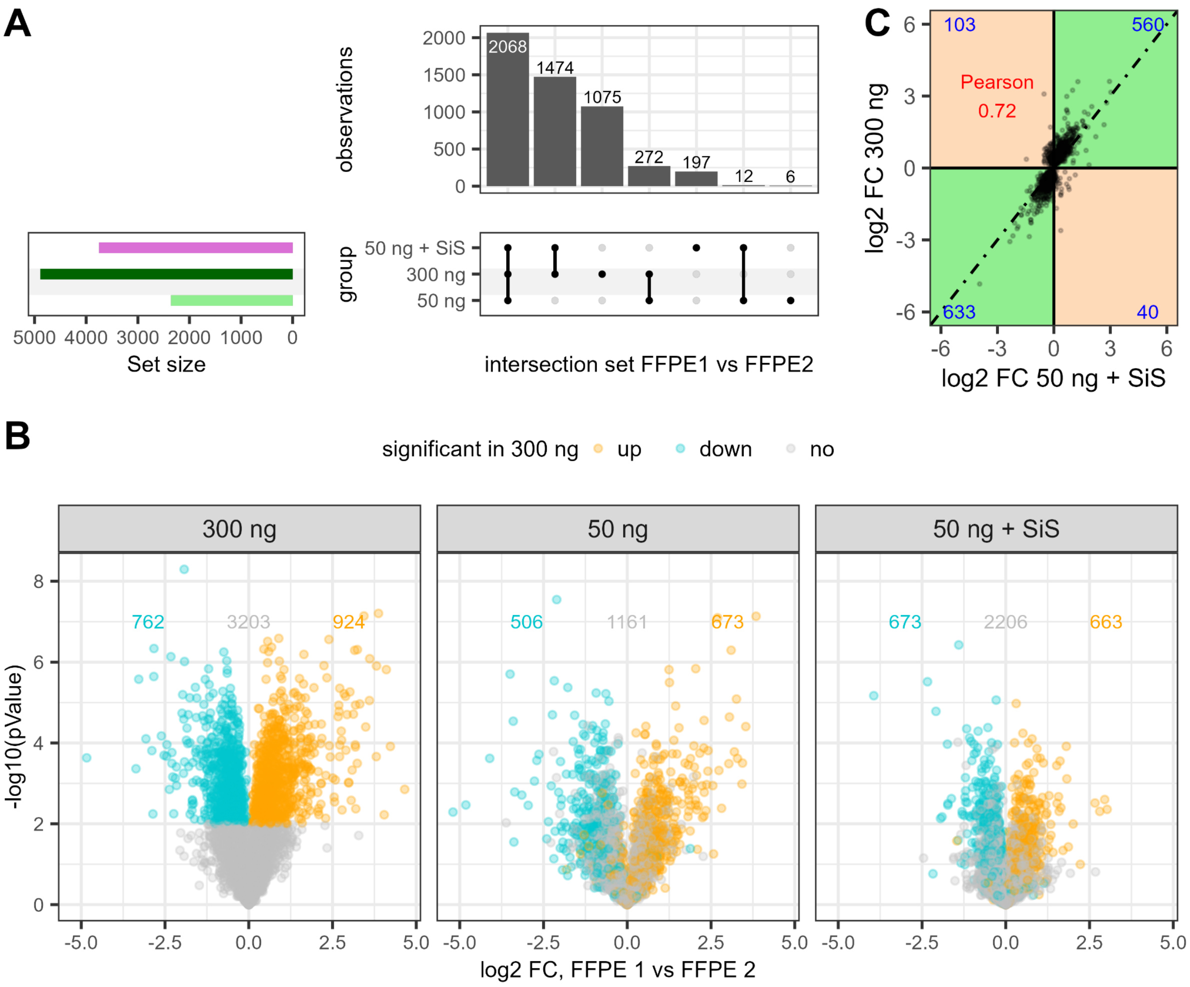
A
observations
2068
1474
1075
272
197
12
6
group
50 ng + SiS
300 ng
50 ng
Set size
intersection set FFPE1 vs FFPE2
C
103
560
633
40
Pearson
0.72
log2 FC 300 ng
log2 FC 50 ng + SiS
B
significant in 300 ng
up
down
no
300 ng
50 ng
50 ng + SiS
762
3203
924
506
1161
673
673
2206
663
-log10(pValue)
log2 FC, FFPE 1 vs FFPE 2

**Figure S13: Application of DIA-SiS (without "requantify") to formalin-fixed paraffin-embedded (FFPE) head and neck squamous cell carcinoma (HNSCC) samples.**

Two FFPE tissue samples (FFPE1 and FFPE2) were compared using different input amounts: 300 ng, 50 ng and 50 ng + 250 ng of the Super-SILAC spike-in reference (50 ng + SiS). **A)** Number of across sample ratios obtained. Reducing the input from 300 ng to 50 ng reduces the number of proteins that can be quantified. Adding the spike-in recovers most of the proteins lost in the low input sample. **B)** Volcano plots for differentially abundant proteins in the FFPE1 vs FFPE2 sample for the different input amounts. Significantly differentially abundant proteins (turquoise and orange) were defined based on the 300 ng input (p-value <= 0.01) and colored accordingly in the other samples. The number of proteins in each subset is indicated. **C)** The correlation of the log2FCs of the differentially abundant protein between the 300 ng input and the 50 ng + ref. input is high. The number of proteins in the quadrants is indicated.

# Pseudocode SILAC Ratio Calculation

## Clean DIA-NN report

1. Filter report.tsv for Precursor.Charge > 1 and remove contaminants

## Create a filter data frame for report.tsv

1. Create an additional column (e.g. "Stripped.Sequence.Charge") that contains the Precursor.Id without the SILAC label information
2. Filter SILAC data on the reference Channel (H) passing
    1. Channel.Q.Value < 0.03
    2. Global.PG.Q.Value < 0.01
    3. For bulk data: Ms1.Translated and Precursor.Translated > 0 and valid value
    4. For single-cell data: Either Ms1.Translated or Precursor.Translated must be > 0 and valid value

   Select relevant columns (Run, Stripped.Sequence.Charge, Protein.Group) and save them in a temporary file ("filterSet")

   For LFQ data: Use Lib.PG.Q.Value < 0.01 and Lib.Q.Value < 0.01 for q-value filtering instead.

3. Extract information which channels - beyond the reference channel – per Stripped.Sequence.Charge pass the filters described in 2)
    1. Inner join filterSet with data (Run, Protein.Group, Precursor.Id, Stripped.Sequence.Charge, Channel.Q.Value & Global.PG.Q.Value, Ms1.Translated, Precursor.Translated)
    2. Filter again as in 2), but over all channels
    3. Group by Run, Stripped.Sequence.Charge, Protein.Group, Channel
    4. Save information which channels that pass the filters ("Channels.Passed.Standard.Filter")
    5. Overwrite temp file "filterSet"
4. Flag on protein level: How many precursors are used per protein group and in which Channels have the precursors passed the filter

    1. Count the number of precursors per Protein.Group and Run in filterSet and save information (includes rescued precursors where only the H channel passed, important for requantify)
    2. Count the number of precursors per protein group and Run in filterSet where all channels passed and save information

5. Return this data frame; it should contain the following information on Protein.Group and Precursor level:

    1. Run
    2. Protein.Group
    3. Stripped.Sequence.Charge
    4. For each precursor, which channels passed the filter
    5. How many precursors per protein in total (includes requantify ratios - only reference channel passed filters)
        6. How many precursors per protein where all channels passed filters

## Calculate Protein.Group SILAC ratios and global intensities

1. Create an additional column (e.g. “Stripped.Sequence.Charge”) that contains the Precursor.Id without the SILAC label information (if not already done in before, see above)
2. Create another column “Channel” that contains the channel information from Precursor.Id
3. Select relevant columns (Run, Protein.Group, Channel, Stripped.Sequence.Charge, Ms1.Translated, Precursor.Translated)
4. Create long table where values from Ms1.Translated and Precursor.Translated are in one column
5. Filter for intensities > 0 and valid, for sc data use any, for bulk require both, Ms1.Translated and Precursor.Translated
6. Create wide table so all intensities are in separate columns per channel
7. Inner join this with filterSet

   1. Filter filterSet prior for required number of precursors per protein to pass (either with or without requantify ratios)
   2. Only keep precursors that passed the corresponding filter set
8. Calculate Protein.Group L/H ratios by
   1. Calculate L/H ratio for every precursor
   2. log10-transform those ratios
   3. Take the median log10 ratios per protein group and run
9. Calculate L protein abundance
   1. Calculate global log10 reference channel abundance
      1. Sum the Ms1.Translated and Precursor.Translated heavy intensities of all precursors of a protein for each sample
      2. log10-transform the summed intensities
      3. Take the median of log10 summed intensities over all samples
   2. Sum (logged) protein L/H ratios and (logged) global H intensities

## Calculate across-sample ratios

1. Make wide table by samples and Protein.Group intensities calculated in 9) (Samples as columns, L SILAC abundances as values)
2. Calculate across-sample ratios: Sample1 - Sample2, …
   1. For LFQ: log10(LFQ Sample 1) – log10(LFQ Sample2)
3. Make sure to not compare a protein where both values only exist thanks to rescued  (“requantify”) ratios
